# Immobilization of Ni(ii) complex on the surface of mesoporous modified-KIT-6 as a new, reusable and highly efficient nanocatalyst for the synthesis of tetrazole and pyranopyrazole derivatives†

**DOI:** 10.1039/d2ra08269a

**Published:** 2023-04-24

**Authors:** Mitra Darabi, Mohsen Nikoorazm, Bahman Tahmasbi, Arash Ghorbani-Choghamarani

**Affiliations:** a Department of Chemistry, Faculty of Science, Ilam University P. O. Box 69315516 Ilam Iran e_nikoorazm@yahoo.com; b Department of Organic Chemistry, Faculty of Chemistry, Bu-Ali Sina University Hamedan 6517838683 Iran

## Abstract

In this paper, KIT-6@SMTU@Ni was successfully synthesized *via* a new method of Ni(ii) complex stabilization on modified mesoporous KIT-6, as a novel and green heterogeneous catalyst. The obtained catalyst (KIT-6@SMTU@Ni) was characterized using Fourier transform infrared spectroscopy (FT-IR), Brunauer–Emmett–Teller (BET) calculation, X-ray diffraction (XRD), atomic absorption spectroscopy (AAS), energy-dispersive X-ray spectroscopy (EDS), X-ray mapping, thermogravimetric analysis (TGA) techniques and scanning electron microscopy (SEM). After complete characterization of the catalyst, it was successfully used for the synthesis of 5-substituted 1*H*-tetrazoles and pyranopyrazoles. Moreover, tetrazoles were synthesized from benzonitrile derivatives and sodium azide (NaN_3_). All tetrazole products were synthesized with high TON, TOF and excellent yields (88–98%) in a reasonable time (0.13–8 h), demonstrating the efficiency and practicality of the KIT-6@SMTU@Ni catalyst. Furthermore, pyranopyrazoles were prepared through the condensation reaction of benzaldehyde derivatives with malononitrile, hydrazine hydrate and ethyl acetoacetate with high TON, TOF and excellent yields (87–98%) at appropriate times (2–10.5 h). KIT-6@SMTU@Ni could be reused for five runs without any re-activation. Significantly, this plotted protocol has prominent benefits, such as applying green solvents, the use of commercially available and low-cost materials, excellent separation and reusability of the catalyst, short reaction time, high yield of products and a facile work-up.

## Abstract 2

Anchoring of Ni(ii) complex onto the surface of mesoporous KIT-6 modified under mild reaction conditions as a highly efficient and reusable nanocatalyst for the synthesis of 5-substituted tetrazoles and pyranopyrazoles.

## Graphical abstract

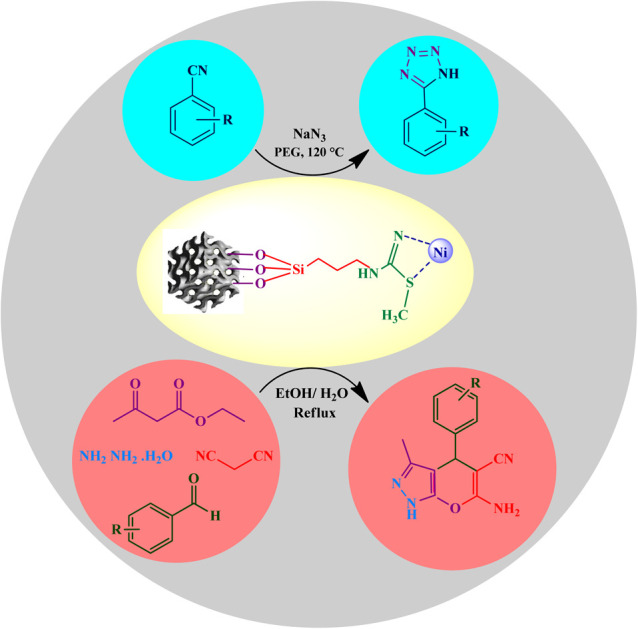

## 1 Introduction

Green chemistry has appeared over the past few decades and has enabled chemists to comprehend these concepts and use them to design advanced syntheses. Chemists have realized the devastating effect of the chemical industry on the environment and human health so that, accordingly, they are involved in trying to minimize it.^1,2^ In this sense, catalysis is one of the principal factors in “green chemistry” and the development of safe environmental catalysts is one of the most important challenges for chemists.^3,4^ Therefore, a stable and “green” catalyst should have clear characteristics, such as high selectivity and activity, low preparation cost, high stability, effective recovery, and reusability. In this regard, heterogeneous catalysts have received outstanding consideration thanks to their extraordinary ability to increase the rates of organic reactions.^1^ In recent years, the stabilization of homogeneous catalysts on solid supports (various nanoparticles) has been extended to the design of heterogeneous catalysts.^2,5^ In this regard, it is worth mentioning that decreasing the particle size would result in increasing its surface area, which would lead to a high capacity for catalyst loading.^6^ Fortunately, to date, various supports, including biochar nanoparticles, zeolites, mesoporous silica materials, iron oxide, carbon nanotubes, metal–organic frameworks, graphene oxide, boehmite nanoparticles, ionic liquids and microporous organic polymers have received a lot of attention for synthesizing heterogeneous catalysts.^7–19^

According to the IUPAC definition, mesoporous materials have pore sizes between 2 and 50 nm. These materials have particular characteristics, such as high surface area, excellent surface performance, orderly porosity, high pore volume, and good mechanical and chemical stability. Furthermore, one known member is mesoporous silica. Mesoporous silica materials are a family of materials that were first discovered in 1992. Among them may be mentioned MCM-48, MCM-41, KIT-1, KIT-6, SBA-15, SBA-16, and MCF-7.^20–27^ Among diverse catalyst supports, KIT-6 has superior benefits, such as an extremely uniform pore distribution, adjustable pore size, dense silanol groups on the surface, great chemical stability, low toxicity, and versatile functionalization chemistry.

Mesoporous materials have received significant consideration due to their unique properties and diverse applications in various fields. In particular, KIT-6 has been studied many times by academics and industries in recent years. KIT-6 is a mesoporous silica with a tridimensional symmetric cubic structure and tridimensional interconnected channels. KIT-6 has a pore diameter of over 6 nm, a wall thickness of 4 to 6 nm, and a bi-continuous interpenetrating network. KIT-6 also has a high surface area and good thermal and mechanical stability.^25,26^ Furthermore, three-dimensional mesoporous materials have the following advantages over two-dimensional opaque rails: (1) they have good ability to absorb large molecules; (2) they have high diffusion indices for the release of reactants; (3) they provide extremely active sites for high uptake; and (4) they prevent pores from clogging.^21^

Considering the pore system, KIT-6 is extremely interconnected consisting of two continuous interpenetrating sub-networks of channels separated by a silica wall. Therefore, the space is divided into two infinite periodic channel networks, unrelated but mutually intertwined.^20^ The synthesis method is an essential parameter that can affect the structure, surface morphology and, eventually, the catalytic efficiency *via* sensitive changes in the particular surface, interaction between different species, and also the distribution and dispersion of active phases on the catalyst. Hydrothermal, co-precipitation, sol–gel, homogeneous precipitation, and impregnation methods are among the customary synthesis methods that have been used.^28–30^ However, KIT-6 has rarely been used as catalytic support in organic chemistry. In this regard the synthesis of aryl tetrazoles, cyclocondensation reactions, oxidation of alcohols, oxidation of sulfides and oxidative coupling of thiols can be mentioned.^31,32^

The synthesis of KIT-6 was reported by Kleitz *et al.*^33^ In the normal hydrothermal synthesis of this material, triblock copolymer Pluronic (P123) is used as the organic structure template, hydrochloric acid is used to maintain the acidity of the medium, butanol is used as a co-solvent and co-template, and tetraethylorthosilicate (TEOS) is the silica source. Moreover, the use of alcohol would greatly affect the organization of the cubic structure and the treatment of the micelles during the synthesis process, facilitating the dissolution of P123. This pattern allows the development of materials with pores of specific morphologies and various sizes.^25,34^

Recently, tetrazoles with a five-membered ring have been given a lot of consideration compared to other N-containing heterocyclic compounds in various fields of science: *i.e.* in organic synthesis, materials science, organometallic chemistry, explosives, photography and recording systems, metallopeptide structures as effective stabilizers, and also in pharmaceutical materials such as antineoplastic, antihypertensive, antibiotic, antiviral, antiallergic and anti-inflammatory drugs. They have also been used as plant growth regulators, receptor modulators, antiviral herbicides, and fungicides.^2,6,35–47^

Multicomponent reactions can be regarded as interesting procedures and powerful methods for the fast synthesis of heterocyclic organic compounds, which include various potentials such as intrinsic convergence, reduction in time, atom economy, savings in cost and energy, environmental benefits, convergence and operational simplicity. For example, pyranopyrazole derivatives can be synthesized from the four-component condensation of hydrazine hydrate, ethyl acetoacetate, aldehyde derivatives, and malononitrile.^48–51^ Significantly, pyranopyrazoles have been used as potential inhibitors of human CHK-1 kinase, and for their insecticidal, pharmaceutical, antimicrobial, anticancer, anti-inflammatory, antiviral, analgesic, and vasodilator activities.^41,52–58^

In this sense, the aim of the present article is to design an effective and convenient method for the stabilization of a new complex of nickel with *s*-methyl isothiouronium sulfate on KIT-6 (KIT-6@SMTU@Ni) in the synthesis of tetrazoles and pyranopyrazoles.

## 2 Experimental

### 2.1 Materials and instruments

All starting materials and solvents employed in this project were purchased from Iranian companies, Merck and Aldrich. The non-ionic surfactant Pluronic P123 triblock copolymer, 3-chloropropyltrimethoxy silane (CPTMS), tetraethylorthosilicate (TEOS) and other chemicals used in the study were purchased from Aldrich and Merck. The catalyst was analyzed using IR spectra of the samples prepared with a KBr disk using a Bruker VERTEX 70 model FT-IR spectrophotometer. X-ray diffraction (XRD) patterns were prepared with a Co radiation source (*λ* = 1.78897 Å) operated at 40 keV. The thermogravimetric analysis (TGA) data were obtained with a Shimadzu DTG-60 analyzer. The morphology was investigated by measuring SEM using a TESCAN MIRA FESEM microscope. The Brunauer–Emmett–Teller (BET) surface area (*S*_BET_) was calculated from the linearity of the BET equation.

### 2.2 KIT-6 synthesis

KIT-6 mesoporous silica was prepared according to published articles.^33,59^ Briefly, Pluronic P123 copolymer (4 g) was added to HCl solution (150 mL, 0.5 mol L^−1^) and then stirred for at least 3 h at 35 °C until complete dissolution. Afterward, *n*-butyl alcohol (4.95 mL) and TEOS (9.2 mL) were injected into the above solution, followed by stirring for 24 h at 35 °C. Subsequently, the mixture was moved into a Teflon-lined autoclave and then heated for 24 h at 100 °C. Finally, the white solid product was filtered without washing, and dried at 100 °C for 12 h. The material was calcined at 550 °C for 4 h.

### 2.3 Modification of KIT-6 with 3-chloropropyltrimethoxysilane (CPTMS)

In this step, a mixture of 1.5 mL of 3-chloropropyltrimethoxysilane and 1.0 g of KIT-6 was refluxed in 40 mL of toluene at 100 °C for 24 h. Afterward, the material was filtered and washed with ethanol and *n*-hexane several times and, finally, dried in an oven at 50 °C to obtain the modified KIT-6 (KIT-6@CPTMS).

### 2.4 Functionalization of KIT-6@CPTMS with *s*-methyl isothiouronium sulfate

1 g of KIT-6@CPTMS powder, 2.6 mmol of ligand (*s*-methyl isothiouronium sulfate) and 5.3 mmol of triethylamine in 40 mL of toluene were stirred under reflux for 24 h at 100 °C. Afterward, the resulting solid (KIT-6@SMTU) was filtered, washed with deionized water and dried at 50 °C for 12 h.

### 2.5 Preparation of nickel catalyst (KIT-6@SMTU@Ni)

**Scheme 1:**
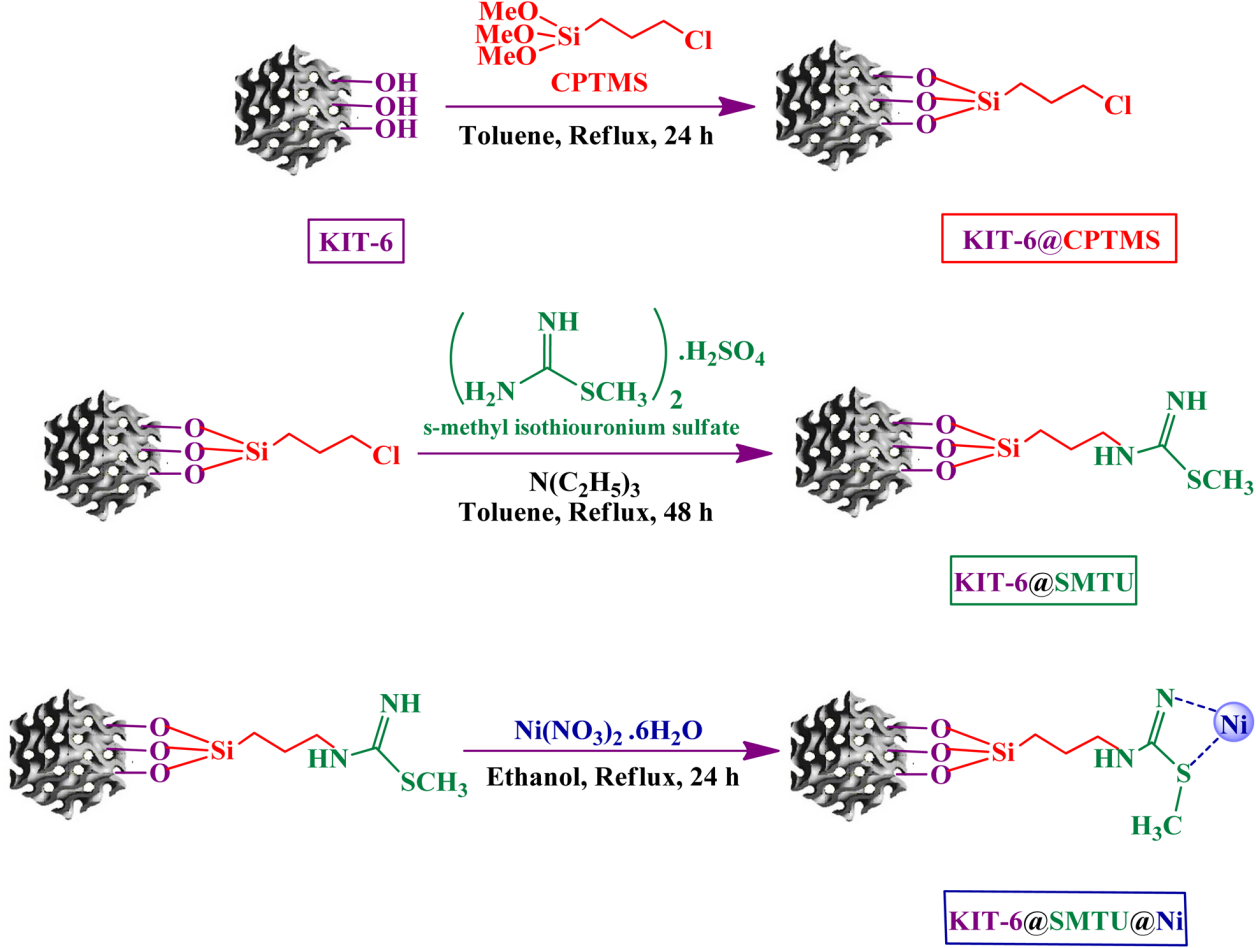
Synthesis of KIT-6@SMTU@Ni.

In the last step, 1.0 g of KIT-6@SMTU was dispersed into 40 mL of ethanol and then 2 mmol of Ni(NO_3_)_2_·6H_2_O was added and, finally, the mixture was refluxed for 24 h at 80 °C. The obtained catalyst was filtered, washed with ethanol and deionized water, and then dried for 12 h at 50 °C to obtain KIT-6@SMTU@Ni (Scheme 1).

### 2.6 Typical procedure for the synthesis of 5-substituted 1*H*-tetrazoles

**Scheme 2:**
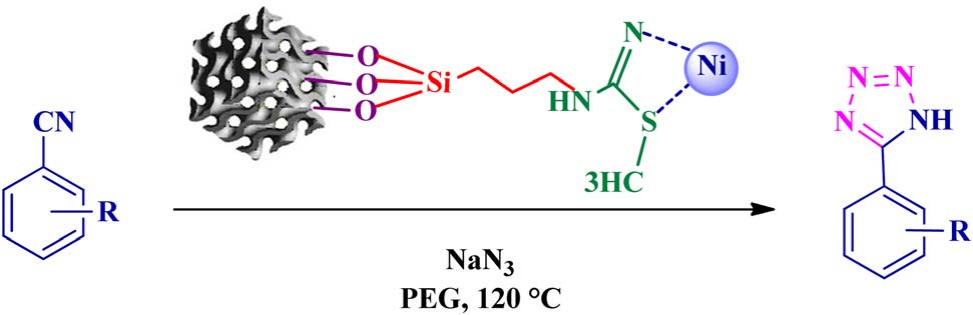
Synthesis of 5-substituted 1*H*-tetrazole derivatives *via* KIT-6@SMTU@Ni.

To synthesize the 5-substituted 1*H*-tetrazole derivatives, sodium azide (1.2 mmol), KIT-6@SMTU@Ni (20 mg, 0.46 mol%), was added to nitrile (1 mmol) and PEG-400 (2 mL) and then the mixture was vigorously stirred at 120 °C. The progression of the reaction was monitored using TLC. After completion of the reaction, the product was isolated by filtration, and then treated with 7 mL of ethyl acetate and 10 mL of HCl (4 N). Finally, the extracted organic phase was dried under air atmosphere (Scheme 2).

### 2.7 Typical procedure for the synthesis of pyranopyrazole derivatives

**Scheme 3:**
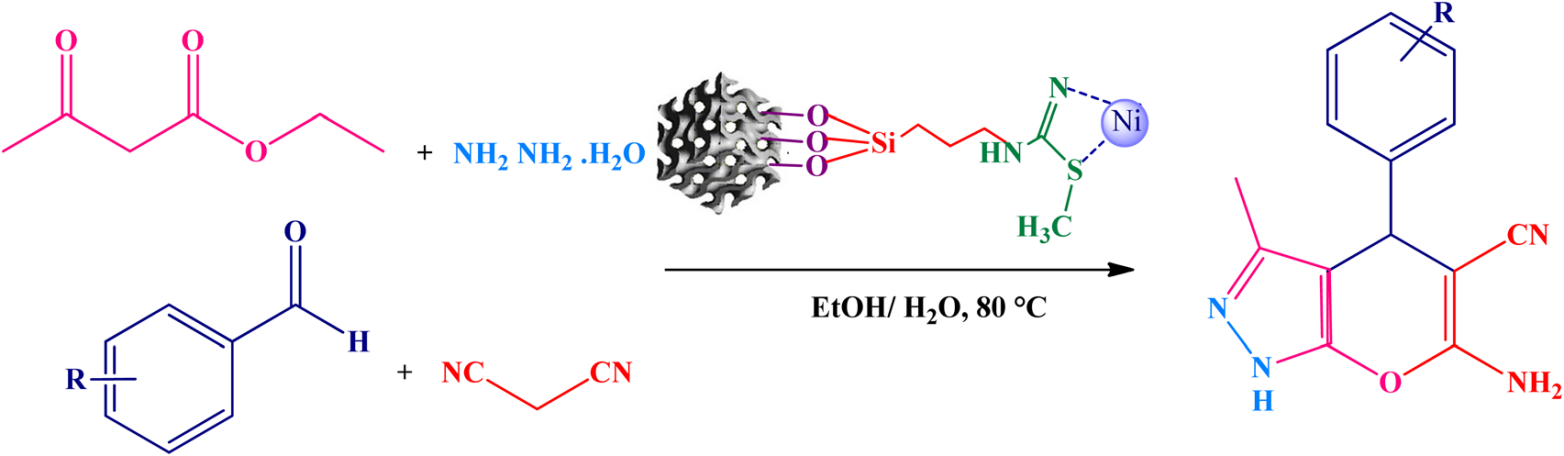
Synthesis of pyranopyrazole derivatives by KIT-6@SMTU@Ni.

The catalytic efficiency of KIT-6@SMTU@Ni was also assessed in the synthesis of pyranoprazoles. A four-component condensation of ethyl acetoacetate (1.0 mmol), hydrazine hydrate (1.0 mmol), aldehyde (1.0 mmol), malononitrile (1.0 mmol), and KIT-6@SMTU@Ni (20 mg, 0.46 mol%) with 1 mL of EtOH : H_2_O (1 : 1) as solvent was conducted in a round-bottom flask (25 mL). The reaction mixture was stirred at 80 °C for different periods. Completion of the reaction process was determined using thin layer chromatography (TLC, acetone : *n*-hexane = 2 : 8). Afterward, dichloromethane was added to the flask; then the catalyst was isolated using simple filtration. Finally, it was dried in air and reused (Scheme 3).

### 2.8 Spectral data

#### 5-(4-Chlorophenyl)-1*H*-tetrazole

^1^H NMR (400 MHz, DMSO_d6_): *δ*_H_ = 8.07–8.04 (d, *J* = 12 Hz, 2H), 7.71–7.68 (d, *J* = 12 Hz, 2H) ppm.

#### 5-(4-Chlorophenyl)-1*H*-tetrazole

^13^C NMR (100 MHz, DMSO_d6_): *δ*_H_ = 164.2, 135.9, 129.6, 128.7, 123.2 ppm.

#### 5-Phenyl-1*H*-tetrazole

^1^H NMR (400 MHz, DMSO_d6_): *δ*_H_ = 8.07–8.01 (m, 2H), 7.65–7.58 (m, 3H) ppm.

#### 5-(4-Nitrophenyl)-1*H*-tetrazole

^1^H NMR (400 MHz, DMSO_d6_): *δ*_H_ = 8.46–8.43 (d, *J* = 12 Hz, 2H), 8.32–8.29 (d, *J* = 12 Hz, 2H) ppm.

#### 2-(1*H*-Tetrazol-5-yl)phenol

^1^H NMR (400 MHz, DMSO_d6_): *δ*_H_ = 8.00–7.97 (d, *J* = 12 Hz, 1H), 7.44–7.38 (t, *J* = 12 Hz, 1H), 7.09–7.06 (d, *J* = 12 Hz, 1H), 7.03–6.98 (t, *J* = 12 Hz, 1H), 3.36 (br, 1H) ppm.

#### 5-(3-Nitrophenyl)-1*H*-tetrazole

^1^H NMR (400 MHz, DMSO_d6_): *δ*_H_ = 8.83 (s, 1H), 8.48–8.46 (d, *J* = 8 Hz, 1H), 8.42–8.40 (d, *J* = 8 Hz, 1H), 7.93–6.87 (t, *J* = 12 Hz, 1H) ppm.

#### 5-(2-Chlorophenyl)-1*H*-tetrazole

^1^H NMR (400 MHz, DMSO_d6_): *δ*_H_ = 16.93 (br, 1H), 7.82–7.79 (d of d, *J* = 8 Hz, 1H), 7.72–7.69 (d, *J* = 8 Hz, 1H), 7.65–7.60 (t of d, *J* = 8 Hz, 1H), 7.58–7.53 (t of d, *J* = 8 Hz, 1H) ppm.

#### 2-(1*H*-Tetrazol-5-yl)benzonitrile

^1^H NMR (400 MHz, DMSO_d6_): *δ*_H_ = 8.11–8.06 (t, *J* = 8 Hz, 2H), 7.95–7.90 (t, *J* = 8 Hz, 1H), 7.81–7.75 (t, *J* = 8 Hz, 1H) ppm.

#### 6-Amino-4-(4-chlorophenyl)-3-methyl-1,4-dihydropyrano[2,3-*c*]pyrazole-5-carbonitrile

^1^H NMR (400 MHz, DMSO_d6_): *δ*_H_ = 12.09 (s, 1H), 7.64–7.52 (m, 2H), 7.40–7.32 (m, 2H), 6.98 (br, 2H), 5.57 (s, 1H), 1.77 (s, 3H) ppm.

#### 6-Amino-4-(4-chlorophenyl)-3-methyl-1,4-dihydropyrano[2,3-*c*]pyrazole-5-carbonitrile

^13^C NMR (100 MHz, DMSO_d6_): *δ*_H_ = 160.9, 154.7, 143.5, 135.7, 131.2, 130.0, 128.5, 120.7, 97.2, 56.7, 35.5, 9.7 ppm.

#### 6-Amino-3-methyl-4-(2-nitrophenyl)-1,4-dihydropyrano[2,3-*c*]pyrazole-5-carbonitrile

^1^H NMR (400 MHz, DMSO_d6_): *δ*_H_ = 12.20 (s, 1H), 7.86–7.84 (d, *J* = 8 Hz, 1H), 7.69–7.64 (t, *J* = 12 Hz, 1H), 7.52–7.46 (t, *J* = 12 Hz, 1H), 7.33–7.31 (d, *J* = 8 Hz, 1H), 7.02 (s, 2H), 5.09 (s, 1H), 1.77 (s, 3H) ppm.

#### 6-Amino-3-methyl-4-(2-nitrophenyl)-1,4-dihydropyrano[2,3-*c*]pyrazole-5-carbonitrile

^13^C NMR (100 MHz, DMSO_d6_): *δ*_H_ = 161.1, 154.9, 149.1, 137.6, 135.7, 131.3, 128.3, 123.6, 120.2, 96.4, 55.9, 31.4, 90.5 ppm.

#### 6-Amino-4-(5-bromo-2-hydroxyphenyl)-3-methyl-1,4-dihydropyrano[2,3-*c*]pyrazole-5-carbonitrile

^1^H NMR (400 MHz, DMSO_d6_): *δ*_H_ = 11.16 (br, 1H), 8.93 (s, 1H), 7.36–7.32 (d, *J* = 16 Hz, 1H), 7.12 (s, 1H), 6.95–6.91 (d, *J* = 16 Hz, 1H), 6.78 (br, 2H), 4.62 (s, 1H), 3.33 (s, 1H), 2.01 (s, 3H) ppm.

#### 6-Amino-4-(3-hydroxyphenyl)-3-methyl-1,4-dihydropyrano[2,3-*c*]pyrazole-5-carbonitrile

^1^H NMR (400 MHz, DMSO_d6_): *δ*_H_ = 12.09 (br, 1H), 9.32 (s, 1H), 7.11–7.06 (d, *J* = 8 Hz, 1H), 6.86 (br, 2H), 6.62–6.58 (m, 2H), 6.52 (s, 1H), 4.47 (s, 1H), 3.36 (br, 1H), 1.81 (s, 3H) ppm.

## 3 Results and discussion

Herein, the preparation and characterization of SMTU@Ni on KIT-6 are reported for the first time. Its application was studied for the synthesis of 5-substituted 1*H*-tetrazoles and pyranopyrazoles as novel heterogeneous and reusable catalysts.

The structure of KIT-6@SMTU@Ni was confirmed using thermogravimetric analysis (TGA), X-ray diffraction (XRD), Fourier transform infrared spectroscopy (FT-IR), atomic absorption spectroscopy (AAS), energy-dispersive X-ray spectroscopy (EDS), scanning electron microscopy (SEM) and Brunauer–Emmett–Teller (BET) calculation.

### 3.1 Low-angle XRD pattern studies

**Fig. 1:**
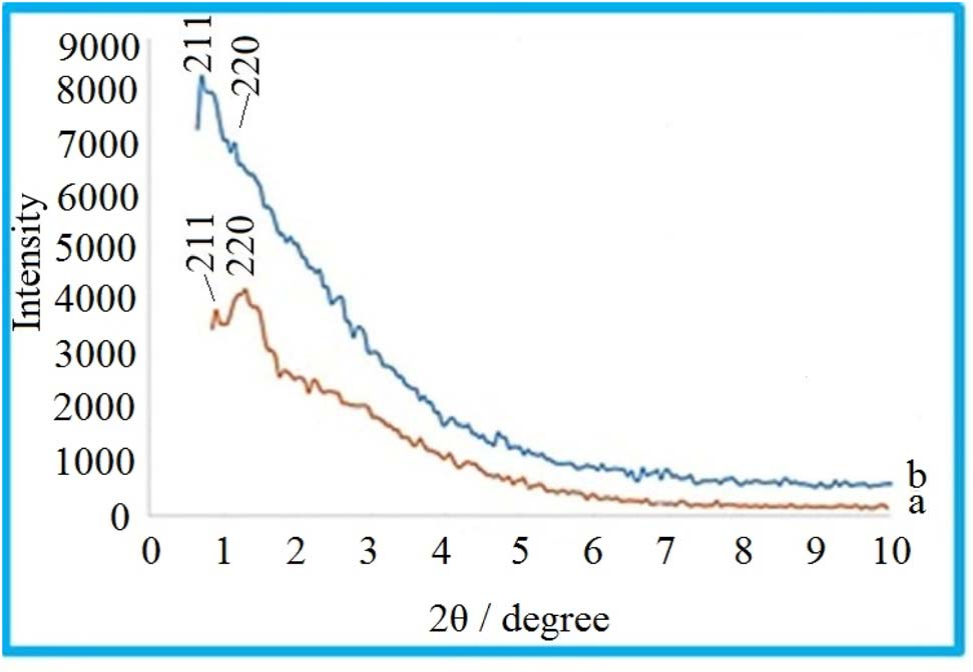
The low-angle XRD patterns of (a) KIT-6 and (b) the KIT-6@SMTU@Ni catalyst.

In order to assess the order of the mesoporous structure of KIT-6, and the material and characterization of the KIT-6@SMTU@Ni catalyst, the samples were characterized using the X-ray diffraction (XRD) method. The low-angle XRD patterns for KIT-6 as a support and the KIT-6@SMTU@Ni catalyst are shown in Fig. 1. It turns out that two peaks of (211) and (220) are recorded for KIT-6 (Fig. 1a), which correspond to the XRD pattern of KIT-6 containing regular cavities with *Ia*3*d* cubic symmetry.^33,59^

The XRD pattern for the catalyst (Fig. 1b) shows that the mesoporous structure of KIT-6 remains well preserved after modification; but the peak intensities are reduced. The decrease in the intensity of the XRD peaks is due to a change in the dispersion pattern and in the pore wall after the functionalization process.

### 3.2 SEM photographs

**Fig. 2:**
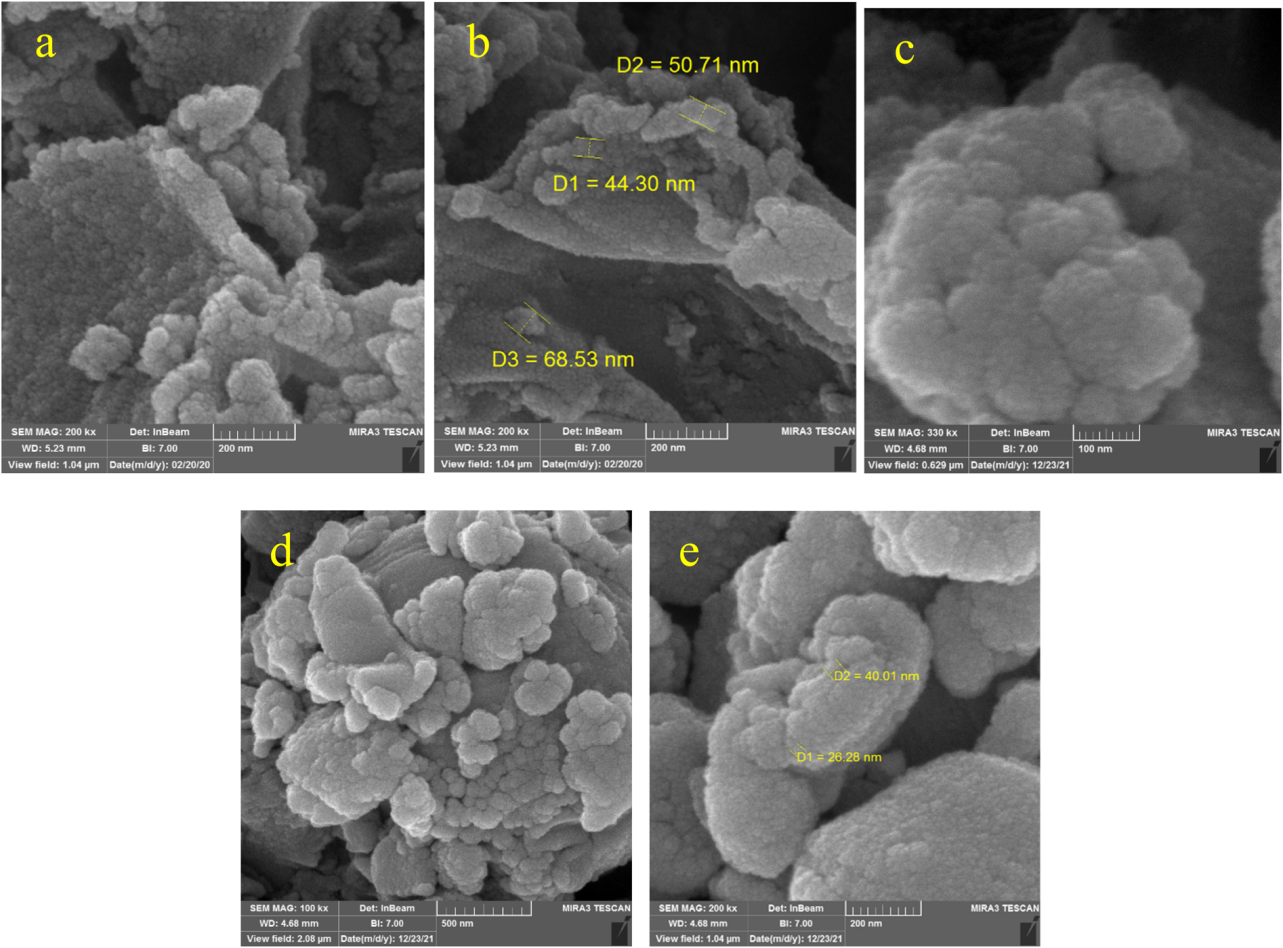
SEM images of (a and b) KIT-6 and (c–e) the KIT-6@SMTU@Ni catalyst.

The SEM technique provides information about a sample, including the topography of the sample, surface properties, shape, size, and placement of particles on the body surface and the composition of the components that make up the sample. In this research, the morphology of the samples was checked by applying the SEM technique. SEM images of KIT-6 and the KIT-6@SMTU@Ni catalyst are shown in Fig. 2. As can be seen, there is no remarkable change in the morphology of the catalyst surface compared to the morphology of KIT-6. This observation confirms that the nickel complex is stabilized in the cavities of KIT-6 and its morphology has not changed. The size of three particles from the KIT-6 support was calculated randomly, and their diameters were in the range of 44.30–68.53 nm. Also, the sizes of two particles from the KIT-6@SMTU@Ni catalyst were calculated randomly, and their diameters were in the range of 26.28–40.01 nm.

### 3.3 Energy dispersive X-ray analysis and elemental mapping

**Fig. 3:**
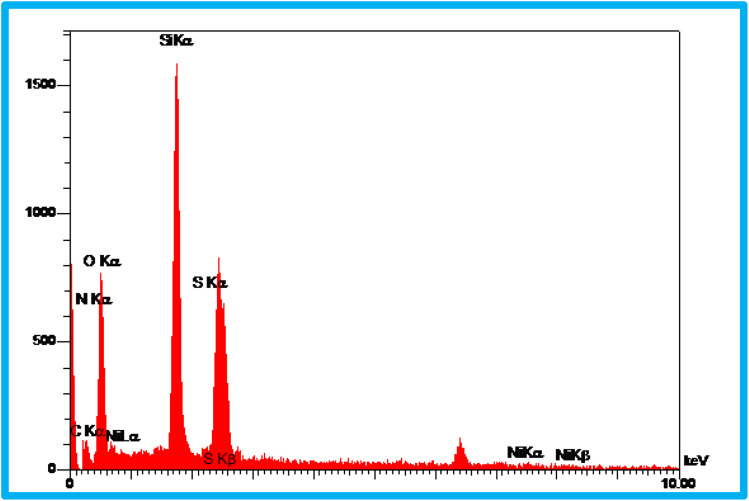
The EDS spectrum of the KIT-6@SMTU@Ni catalyst.

**Fig. 4:**
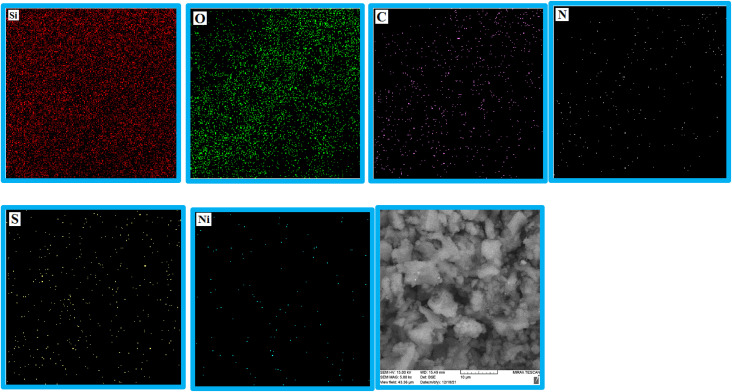
Elemental mapping images of the KIT-6@SMTU@Ni catalyst.

EDS analysis was undertaken to show the presence of elements in the structure of the KIT-6@SMTU@Ni catalyst (Fig. 3). As depicted, the EDS result of this catalyst (KIT-6@SMTU@Ni) shows the presence of silicon, oxygen, carbon, nitrogen, and also nickel species. Moreover, the elemental X-ray mapping of the catalyst (KIT-6@SMTU@Ni) confirmed that the elements (oxygen, carbon, silicon, nitrogen, and Ni) are distributed homogeneously on the catalyst surface (Fig. 4). These results indicate that the nickel complex has been successfully immobilized on the KIT-6 support.

Moreover, the exact amount of Ni which was loaded on KIT-6 was calculated using AAS analysis (0.23 × 10^−3^ mol g^−1^).

### 3.4 Thermogravimetric analysis studies

**Fig. 5:**
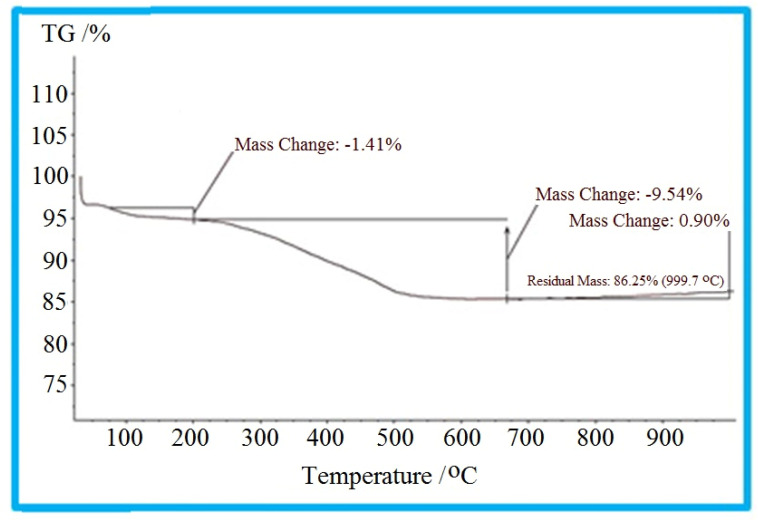
TGA curve of the KIT-6@SMTU@Ni catalyst.

Graphs from TGA analysis show the change in mass of the sample based on a function of temperature where different molecules are adsorbed by heat at different temperatures. As illustrated in the TGA diagram of the KIT-6@SMTU@Ni catalyst (Fig. 5), the weight decrease from 35 °C to 200 °C can be assigned to the removal of adsorbed organic solvents and water in the mesoporous materials.^60,61^ The weight loss observed in the temperature range of 200 °C to 700 °C can be attributed to the disintegration of the immobilized organic compounds. These results indicate that the organic groups have been successfully stabilized on the KIT-6 surface.

### 3.5 FT-IR spectra

**Fig. 6:**
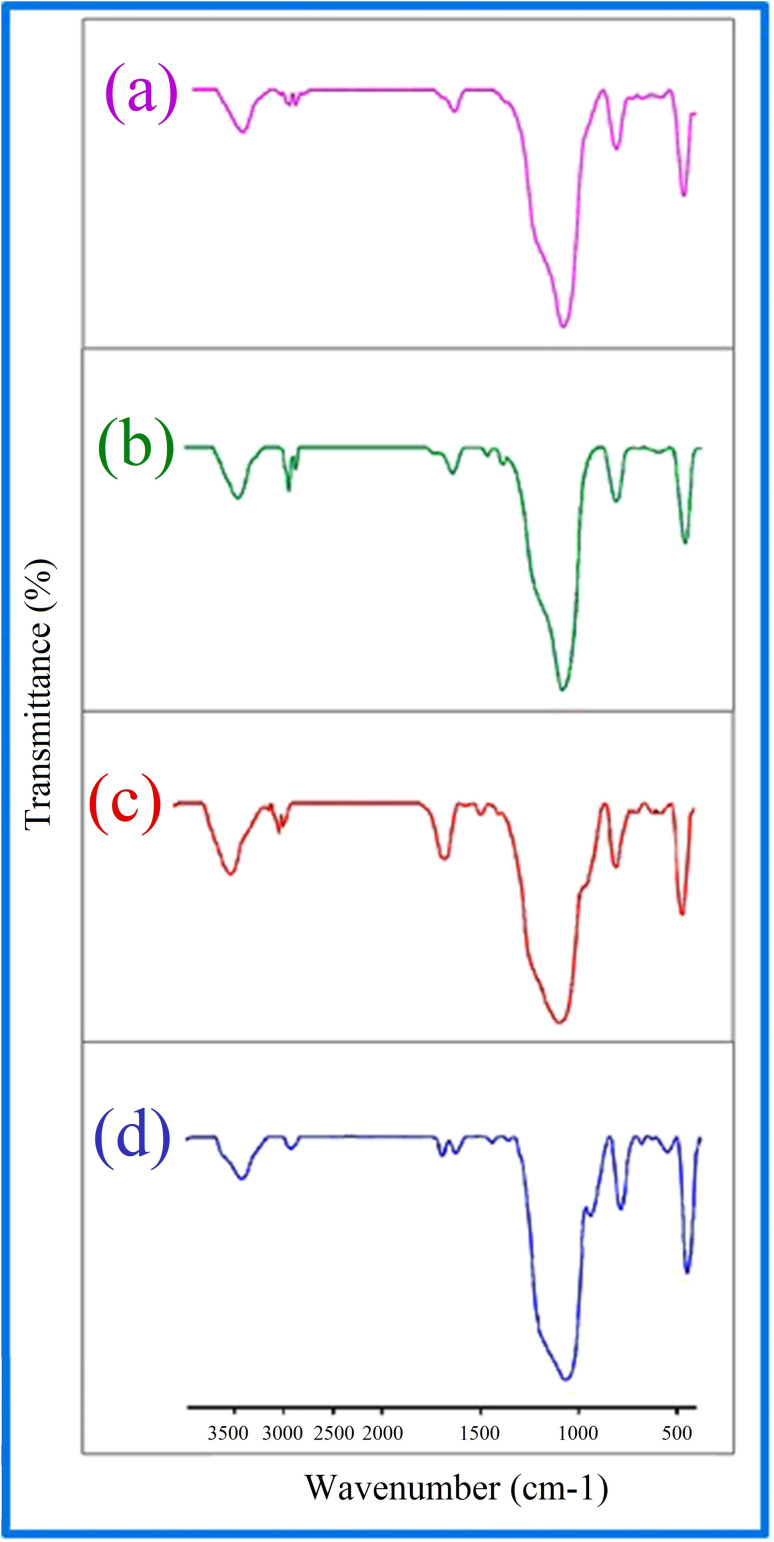
FT-IR spectra of (a) KIT-6, (b) KIT-6@Cl, (c) KIT-6@SMTU, (d) KIT-6@SMTU@Ni catalyst.

The type of functional groups present in KIT-6 and the KIT-6@SMTU@Ni catalyst can be illustrated by FT-IR spectroscopy. In the FT-IR spectrum for KIT-6 nanostructures (Fig. 6), a broad band in the 3445 cm^−1^ region is relevant to the stretching vibrations and the absorption spectrum at about 1637 cm^−1^ is related to the flexural vibrations of the surface OH groups.^62^ The absorption band is at about 1079 cm^−1^ for the asymmetric stretching vibrations of the Si–O–Si groups. The absorption spectra of the symmetric stretching vibrations of the Si–O–Si groups are observed in the range of 807 cm^−1^. The peak observed in the 462 cm^−1^ region is related to the bending vibrations of the Si–O–Si groups.^59^ After the functionalization of KIT-6 with 3-chloropropyltrimethoxysilane (CPTMS), new peaks appear. The existence of anchored CPTMS is confirmed *via* the C–H stretching vibrations at 2925 cm^−1^.^63,64^ Curve (c) shows bands at 1470 cm^−1^ due to C–C, and at 1648 cm^−1^ due to C=N, which proves that the *s*-methyl isothiouronium sulfate (SMTU) molecules have been bonded on the KIT-6@CPTMS surface. The signal of the C=N functional group shifted from 1648 cm^−1^ to 1636 cm^−1^ in the FT-IR spectrum of KIT-6@SMTU@Ni. This change is assigned to the coordination of *s*-methyl isothiouronium with Ni nanoparticles.^36,61,64^ This result indicates that the Ni nanoparticles were successfully immobilized on KIT-6.

### 3.6 N_2_ adsorption–desorption isotherm studies

The nitrogen adsorption method is a very valuable method to determine the physical properties of materials. This technique is generally used to determine the area, volume, and diameter of pores, describing the size distribution of pores of mesostructured materials.

**Fig. 7:**
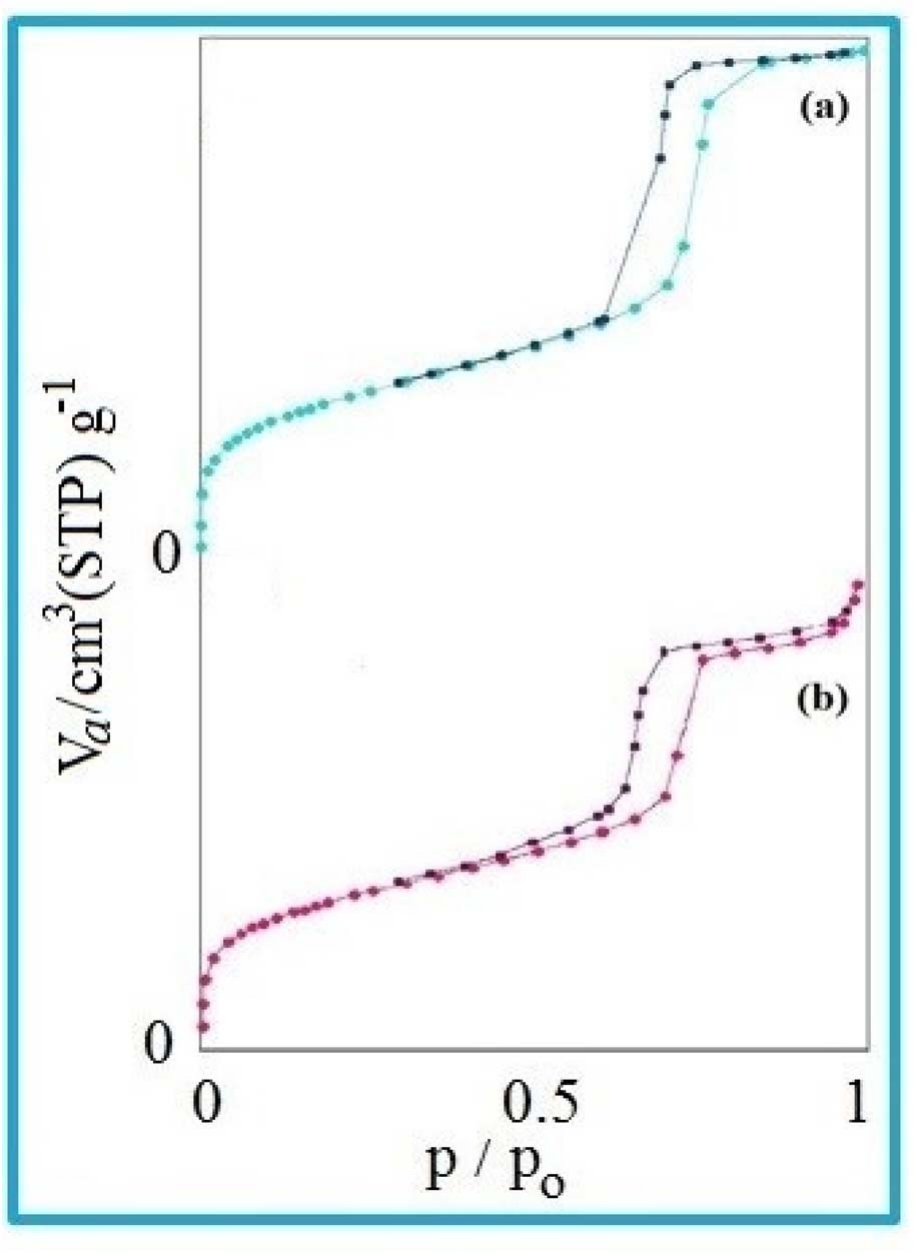
N_2_ adsorption–desorption of (a) KIT-6 and (b) the KIT-6@SMTU@Ni catalyst.

**Fig. 8:**
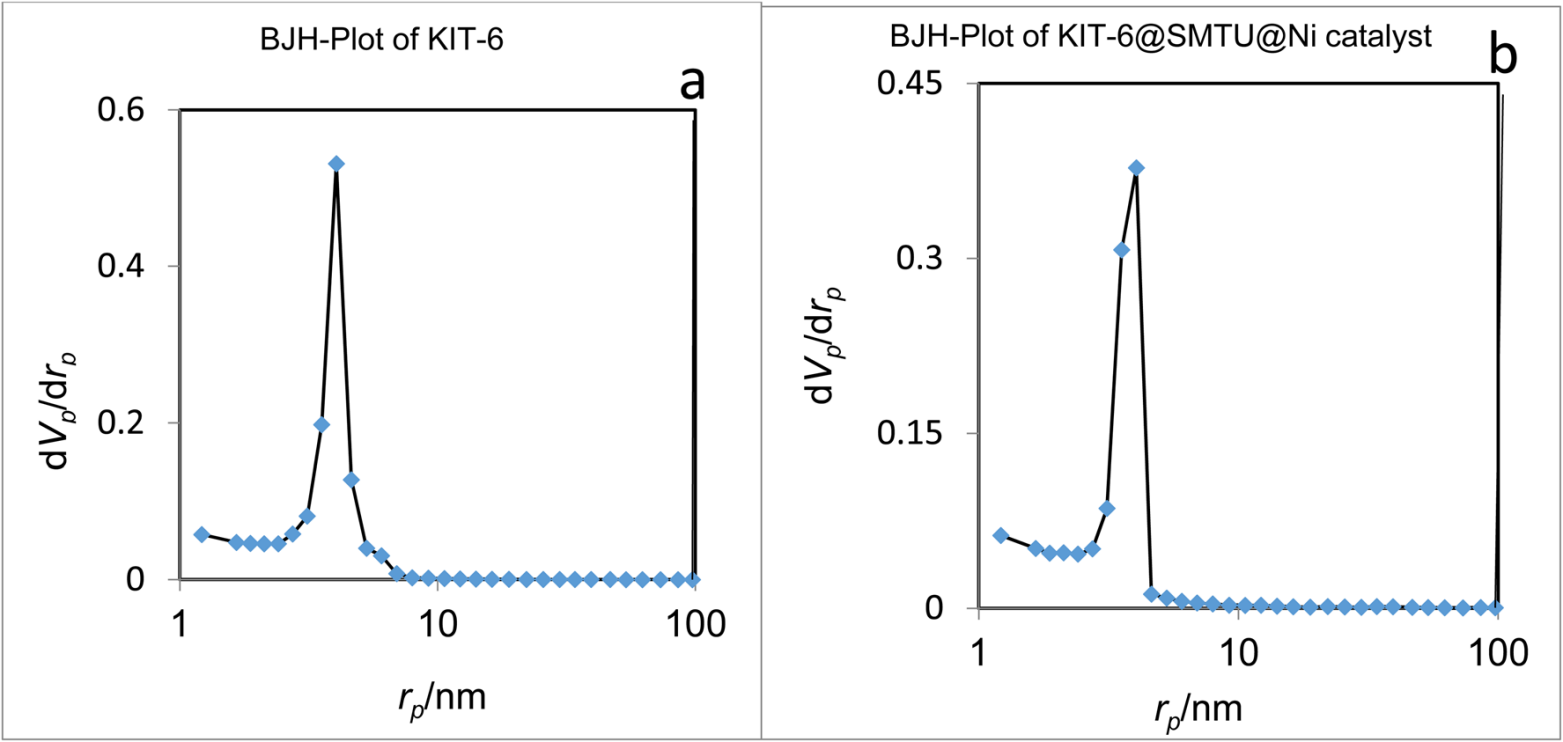
BJH diagrams of KIT-6 (a) and the KIT-6@SMTU@Ni catalyst (b).

For the nitrogen adsorption–desorption isotherms, type IV isotherm distributions with an H1 hysteresis loop for the mesoporous KIT-6 material and KIT-6@SMTU@Ni catalyst are depicted in Fig. 7.^65,66^ Also, BJH diagrams of KIT-6 and the KIT-6@SMTU@Ni catalyst are shown in Fig. 8. Both patterns prove the existence of mesoporous materials and show the uniformity of the synthesized mesoporous KIT-6 and catalyst. Moreover, the stability of the pattern in the functionalized KIT-6 shows that the KIT-6 structure is well preserved after functionalization. Besides, the stabilization of the Ni-complex does not change in the structure of KIT-6. Nitrogen adsorption–desorption data, indicating the specific surface area (581.14 m^2^ g^−1^), pore volume (0.7734 cm^3^ g^−1^) and average pore diameter (5.32 nm), for the used KIT-6 support and the prepared catalyst, are given in Table 1. As shown in the table, the data for surface area and pore volume for KIT-6@SMTU@Ni decreased compared to KIT-6 due to loading of the SMTU@Ni complex in the KIT-6 pores.

**Table 1:** Structural and textural parameters of KIT-6 and KIT-6@SMTU@Ni samples

Sample	*S*_BET_ (m^2^ g^−1^)	Total proven volume (cm^3^ g^−1^)
KIT-6	581.14	0.7734
KIT-6@SMTU@Ni	509.65	0.6965

In fact, modifying the surface of KIT-6 reduces the space of the pore, which changes the volume and surface area of the pore. However, the regular structure of the pores is preserved in the composition of the initial mesoporous KIT-6 after surface correction.

### 3.7 Catalytic studies

After the catalyst synthesis and identification, in order to investigate the catalytic activity of KIT-6@SMTU@Ni as a recoverable catalyst, we employed it for the synthesis of tetrazoles (Scheme 2) and pyranopyrazoles (Scheme 3). Optimization of the reaction conditions for the synthesis of 5-substituted 1*H*-tetrazoles (considering the solvent effect, amount of catalyst, and temperature) was performed for the reaction of benzonitrile to the corresponding tetrazole as a model reaction. Before optimizing the temperature and amount of the catalyst used, it is necessary to select a suitable solvent; therefore, several solvents, such as dioxin, DMF, PEG, H_2_O, and DMSO, were used. The results showed that PEG-400 can be a suitable solvent for the reaction, which can provide the conditions for the reaction in a shorter time and higher efficiency. Subsequently, the efficacy of the amount of catalyst on the rate of progression was also investigated. Moreover, the effect of temperature on the reaction rate was also investigated. It was observed that the reaction progressed well with 20 mg of catalyst at 120 °C. Therefore, 1.2 mmol of sodium azide, 1 mmol of benzonitrile, 20 mg of KIT-6@SMTU@Ni catalyst (0.46 mol%), and PEG solvent at 120 °C were selected as the best reaction conditions (Table 2, entry 2). To evaluate the efficiency of this synthetic method, various derivatives of tetrazoles were synthesized by reacting various nitriles with sodium azide. These results are summarized in Table 3.

**Table 2:** Effect of various parameters for the synthesis of 5-substituted 1*H*-tetrazoles in the presence of the KIT-6@SMTU@Ni catalyst

Entry	Solvent	Temp. (°C)	Catalyst (mg)	Time (h)	Yield (%)
1	PEG	120	10	4.92	90
2	PEG	120	20	3	94
3	PEG	120	30	2.63	92
4	PEG	100	20	3.25	63
5	PEG	80	20	3.33	59
6	DMSO	120	20	3.58	78
7	DMF	120	20	4	81
8	Dioxan	120	20	8.5	36
9	H_2_O	100	20	8.08	43

**Table 3:** Preparation of 5-substituted 1*H*-tetrazoles in the presence of KIT-6@SMTU@Ni

Entry	Substrate	Product	Time (h)	Yield^a^ (%)	TON	TOF (h^−1^)
1	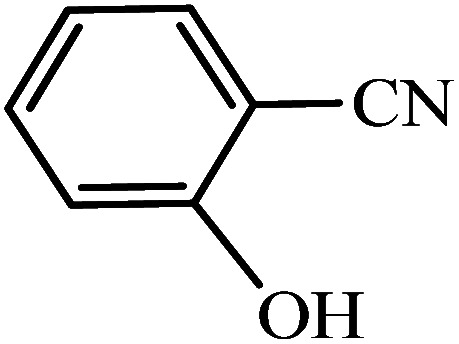	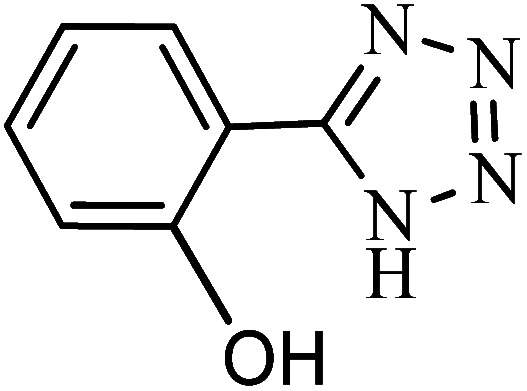	0.5	98	213	426
2	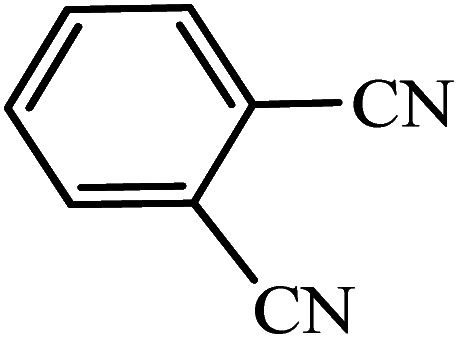	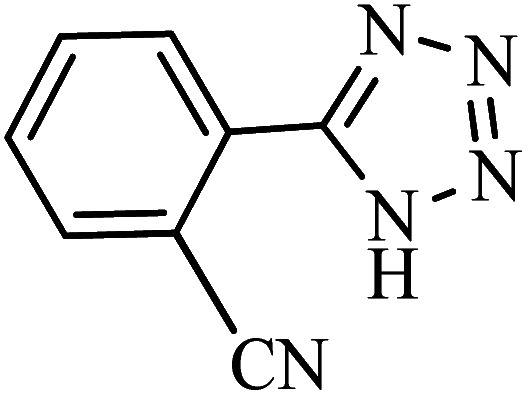	1	96	209	209
3	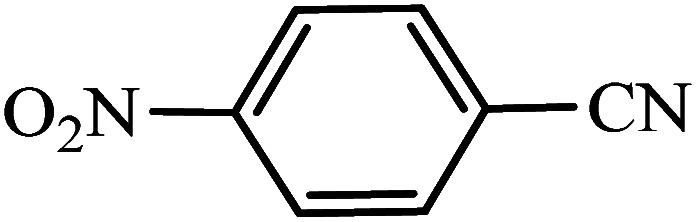	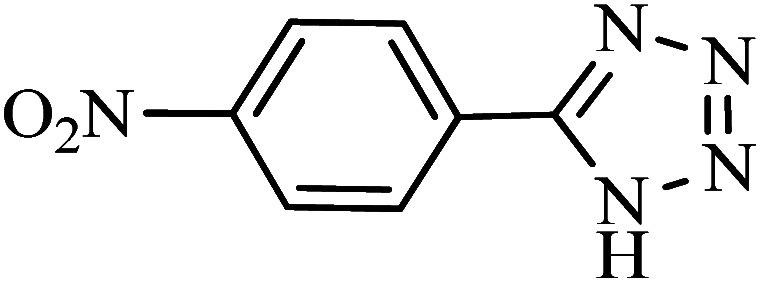	8	88	191	23.9
4	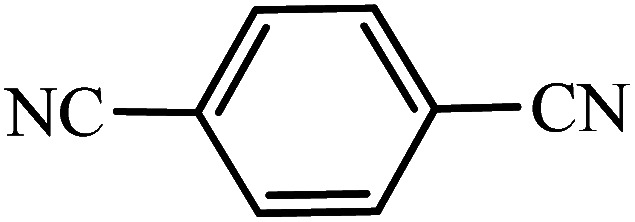	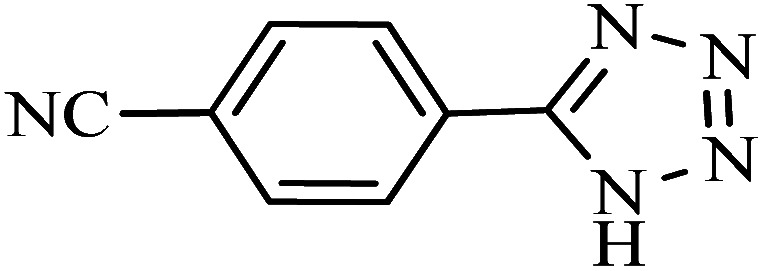	0.5	98	213	426
5	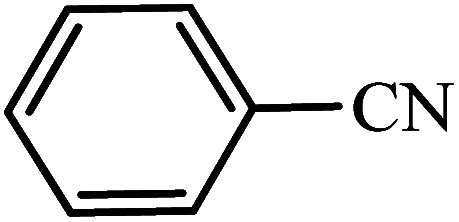	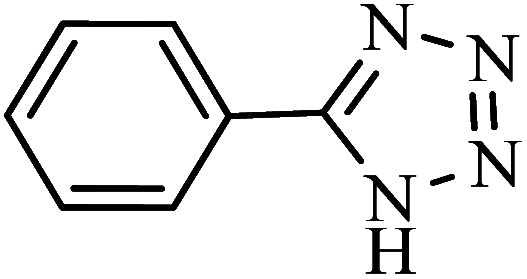	3	95	206	68.7
6	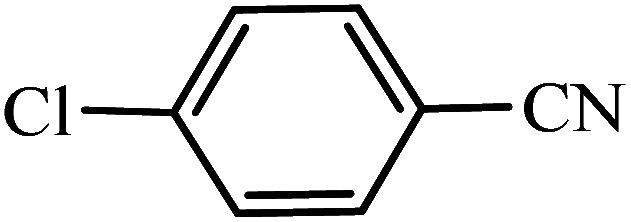	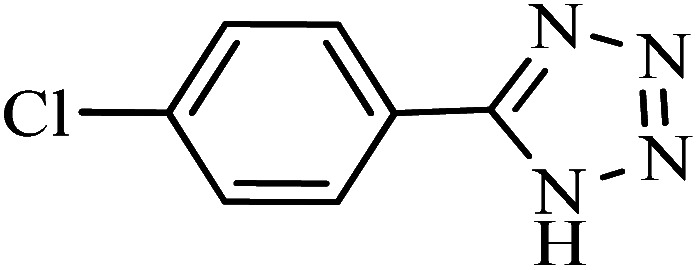	5	90	196	39.2
7	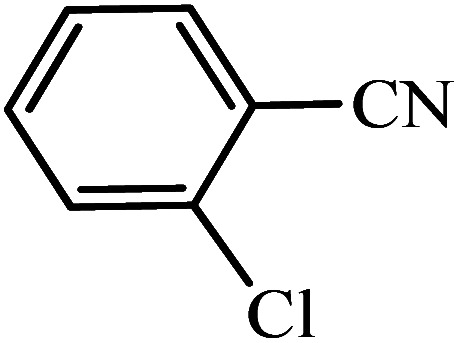	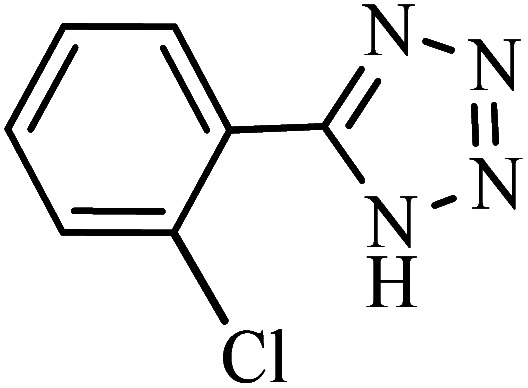	5	93	202	40.4
8	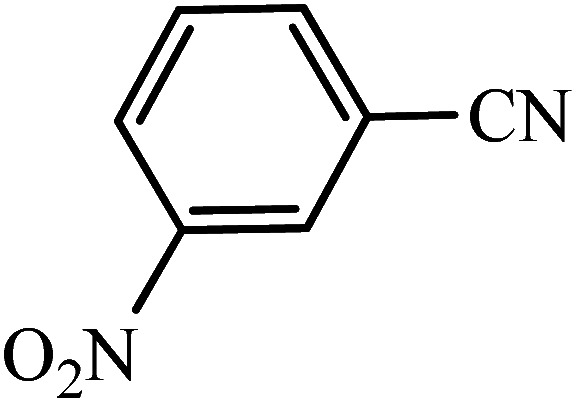	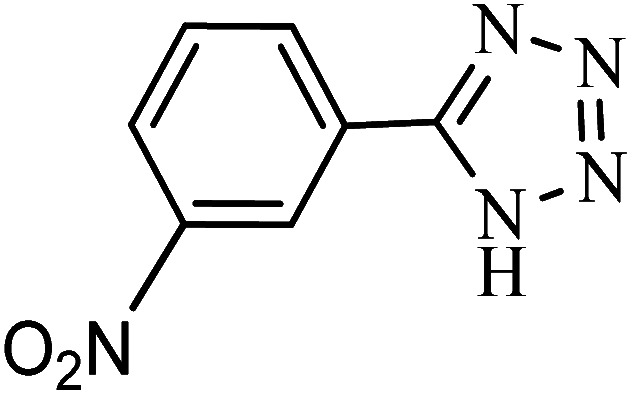	5	89	193	38.6
9	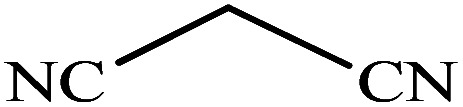	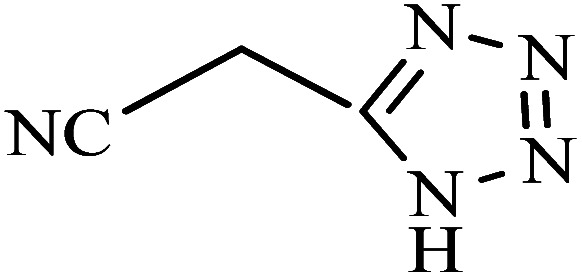	0.5	95	206	412
10	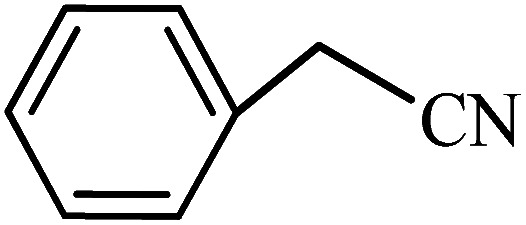	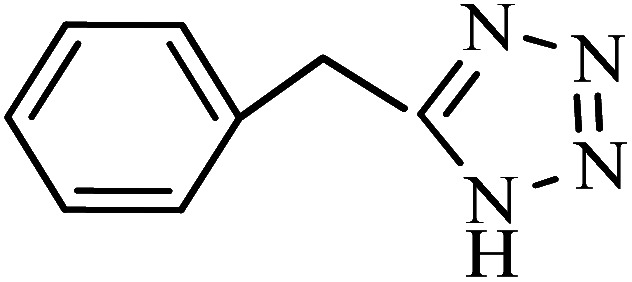	2.5	96	209	83.6

a Reaction conditions: KIT-6@SMTU@Ni catalyst (20 mg, 0.46 mol%), benzonitrile (1 mmol), sodium azide (1.2 mmol) and PEG solvent at 120 °C.

**Scheme 4:**
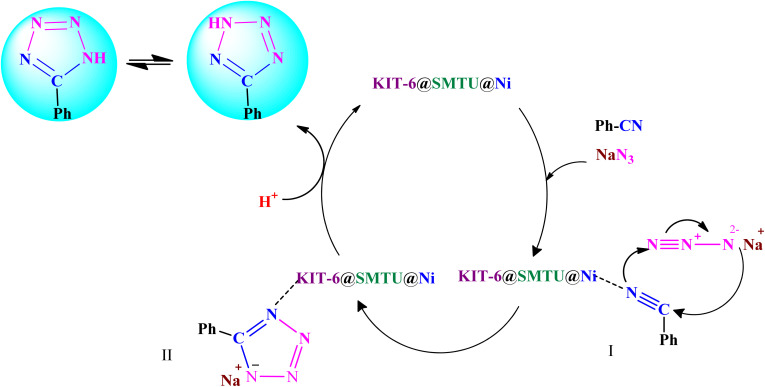
The proposed mechanism for the synthesis of tetrazoles in the existence of KIT-6@SMTU@Ni.

A reaction mechanism for the synthesis of 5-substituted 1*H*-tetrazoles is shown in Scheme 4. As shown in Scheme 4, the nitrogen atom of the nitrile primarily coordinates to the metal (Ni) of the catalyst, to pull the π electron density onto the N atom and make it more nucleophilic. This interaction forms intermediate I. Indeed, KIT-6@SMTU@Ni acts as a Lewis acid, which activates the nitrile groups *via* coordination. Afterwards, it reacts with sodium azide to form intermediate II. The protonolysis produces tetrazole as the final product, and the catalyst is released for the next run of the reaction.^67–69^

It should be noted that there are different methods for preparing pyranopyrazoles; but most of these methods have limitations such as incompatibility with the environment, reaction time, high cost, production of by-products, purification problems, selectivity, and low productivity of products. Therefore, it will always be important to provide methods that can solve these problems.

To optimize the reaction conditions for the synthesis of pyranopyrazoles, diverse parameters, such as the amount of catalyst and different solvents in the four-component concentrations of malononitrile (1 mmol), 4-chlorobenzaldehyde (1 mmol), ethyl acetoacetate (1 mmol) and hydrazine hydrate (1 mmol) were investigated as the model reaction (Table 3). In order to select the appropriate solvent for the synthesis of pyranopyrazoles using the KIT-6@SMTU@Ni catalyst, the 4-chlorobenzaldehyde reaction was investigated as a sample reaction in the presence of a constant amount of catalyst at various temperatures using various solvents, such as water, ethanol and PEG. It should be noted that among the various solvents, water : ethanol (1 : 1) solvent in equal proportions was selected as a green solvent with low toxicity as the best solvent for the preparation of pyranopyrazole derivatives. To investigate the efficacy of the catalyst on the yield and other reaction conditions, the model reaction was done using various amounts of catalyst at 80 °C. Significantly, hydrazine hydrate (1 mmol), 4-chlorobenzaldehyde (1 mmol), malononitrile (1 mmol), ethyl acetoacetate (1 mmol), 20 mg of KIT-6@SMTU@Ni catalyst (0.46 mol%) and water : ethanol (1 : 1) solvent in equal proportions at 80 °C were selected as the best reaction conditions (Table 4, entry 2). After optimizing the reaction conditions and in order to expand the scope of application of this method, various types of pyranopyrazole were synthesized using various derivatives of aldehydes in the presence of KIT-6@SMTU@Ni catalyst in water : ethanol (1 : 1) solvent in equal proportions at 80 °C (Table 5).

**Table 4:** Reaction between hydrazine hydrate, 4-chlorobenzaldehyde, malononitrile and ethyl acetoacetate catalyzed by KIT-6@SMTU@Ni in various solvents under reflux conditions

Entry	Solvent	Temp. (°C)	Catalyst (mg)	Time (h)	Yield (%)
1	EtOH : H_2_O	80	10	7	87
2	EtOH : H_2_O	80	20	4	90
3	EtOH : H_2_O	80	30	4.5	91
4	PEG	80	20	8	47
5	EtOH	80	20	8	65
6	H_2_O	80	20	4.5	61

**Table 5:** Catalytic efficiency of KIT-6@SMTU@Ni in the synthesis of pyranopyrazoles^a^

Entry	Substrate	Product	Time (h)	Yield (%)	TON	TOF (h^−1^)
1	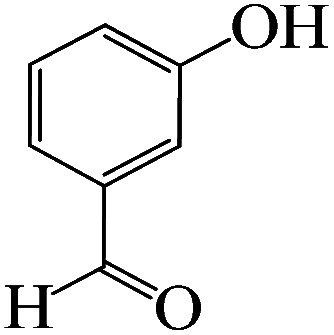	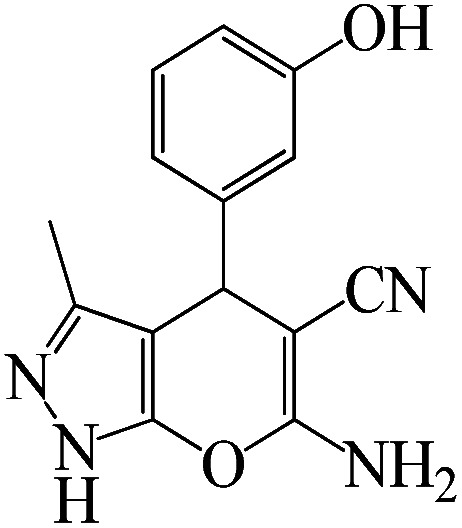	5	98	213	42.6
2	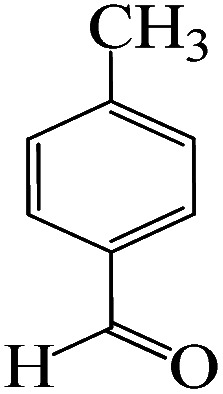	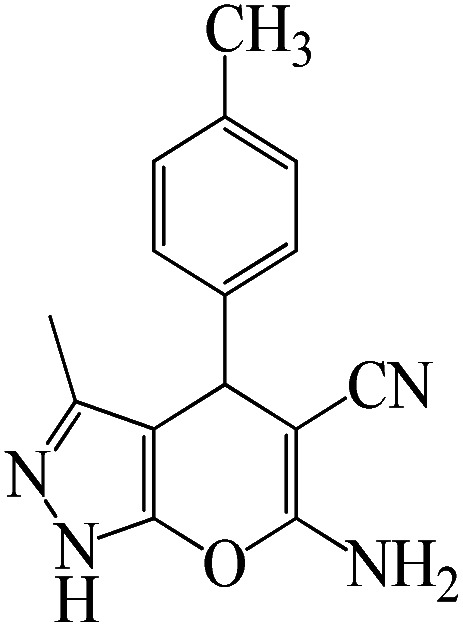	7	90	196	28
3	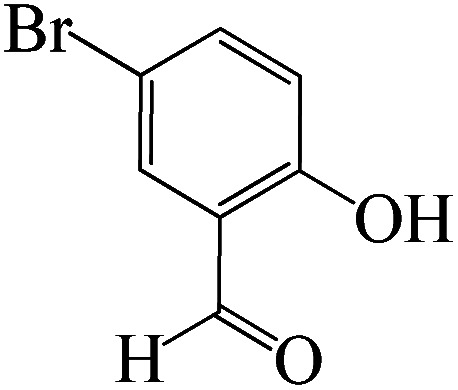	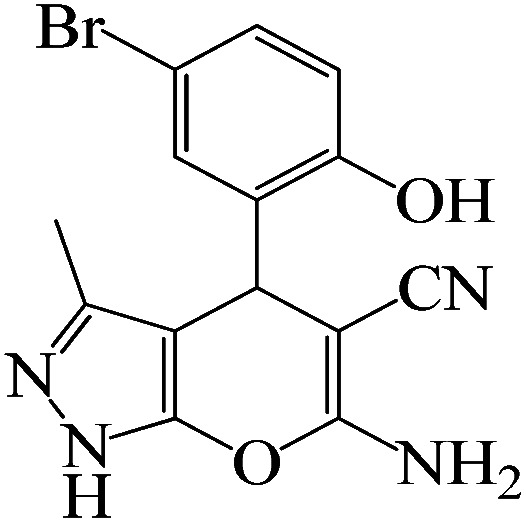	2	96	209	104.5
4	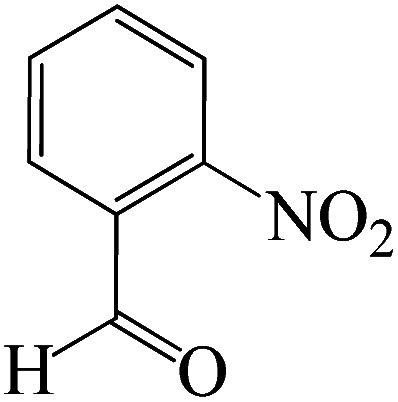	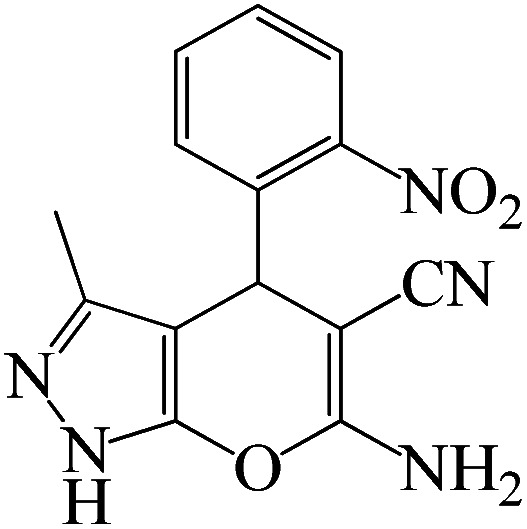	7	88	191	27.3
5	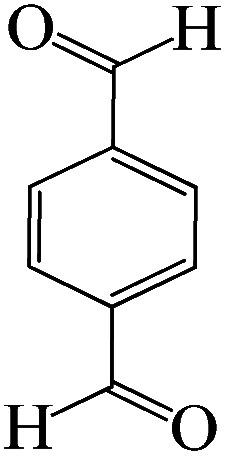	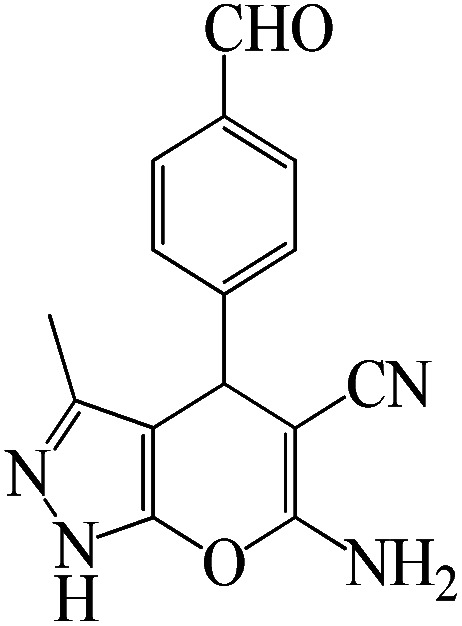	2	93	202	101
6	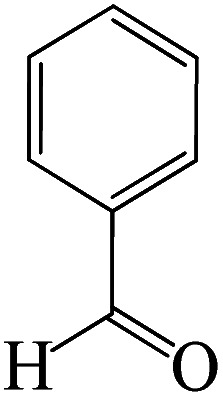	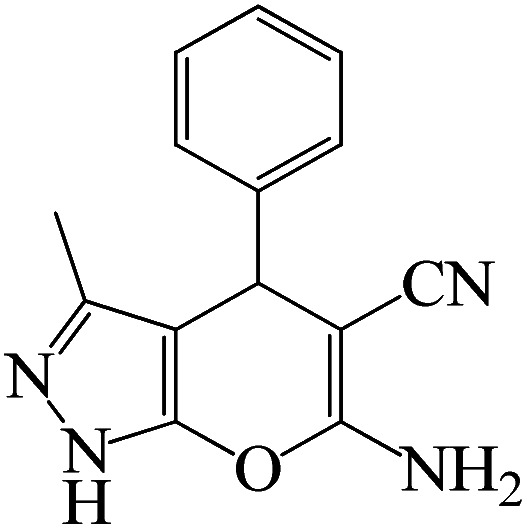	5.5	91	198	36
7	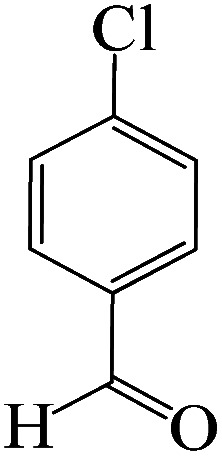	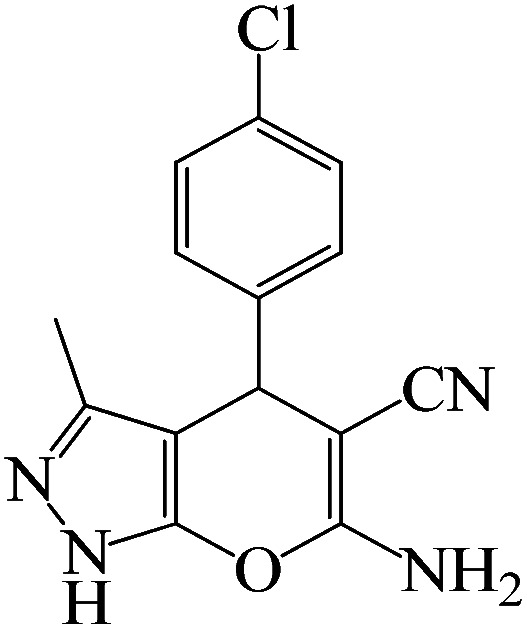	4	90	196	49
8	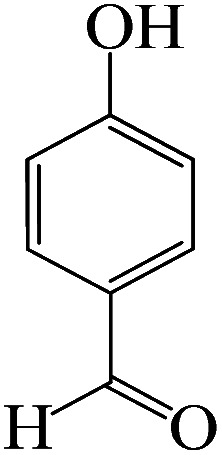	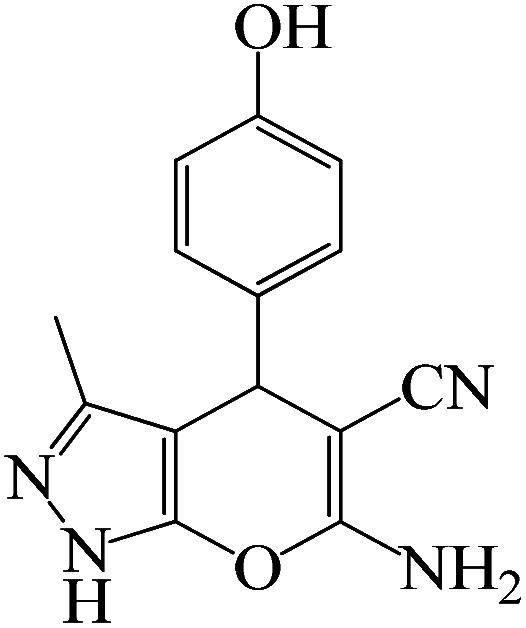	9	92	200	22.2
9	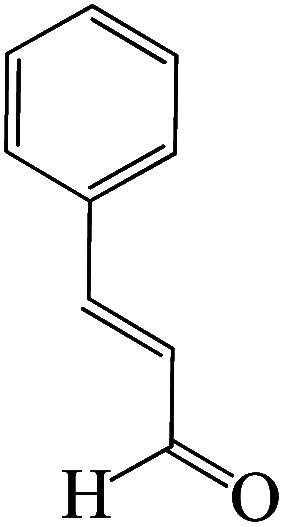	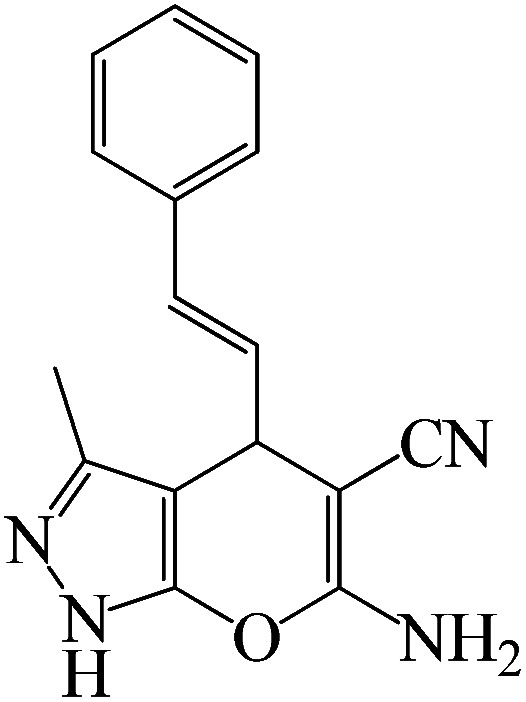	10.5	87	189	18

a Reaction conditions: hydrazine hydrate (1 mmol), benzaldehyde (1 mmol), malononitrile (1 mmol), ethyl acetoacetate (1 mmol), KIT-6@SMTU@Ni catalyst (20 mg, 0.46 mol%) and water : ethanol (1 : 1) solvent at 80 °C.

**Scheme 5:**
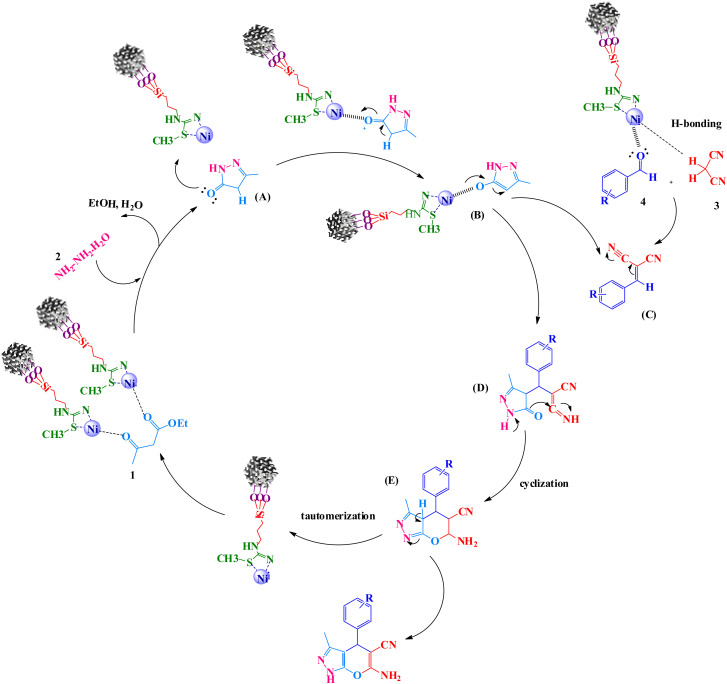
The proposed mechanism for the preparation of pyranopyrazoles in presence of KIT-6@MSTU@Ni.

A reaction mechanism for the formation of pyranopyrazoles is suggested in Scheme 5. Primarily, the KIT-6@SMTU@Ni catalyst activated the carbonyl groups of ethyl acetoacetate. Afterward, the carbonyl groups of ethyl acetoacetate were exposed to nucleophilic attack by (NH_2_ groups of) hydrazine hydrate with two nucleophilic sites. The intermediate pyrazolone A was produced while eliminating water and ethanol molecules. Subsequently, intermediate A transferred a pair of electrons on the oxygen atom of the carbonyl group under Ni^2+^ (KIT-6@SMTU@Ni catalyst) to give the enol form of pyrazolone ring B. In the next step, a Knoevenagel condensation was formed by activating the carbonyl group of the aldehyde 4 and the methylene group of malononitrile 3 with H-bonding by the catalyst to give intermediate C. Then, a Michael addition reaction between the catalyst-activated intermediate B and C resulted in intermediate D, which underwent intramolecular cyclization, providing intermediate E. Finally, through tautomerization of intermediate E, the desired products were obtained.^70^

### 3.8 Reusability study of the catalyst

**Fig. 9:**
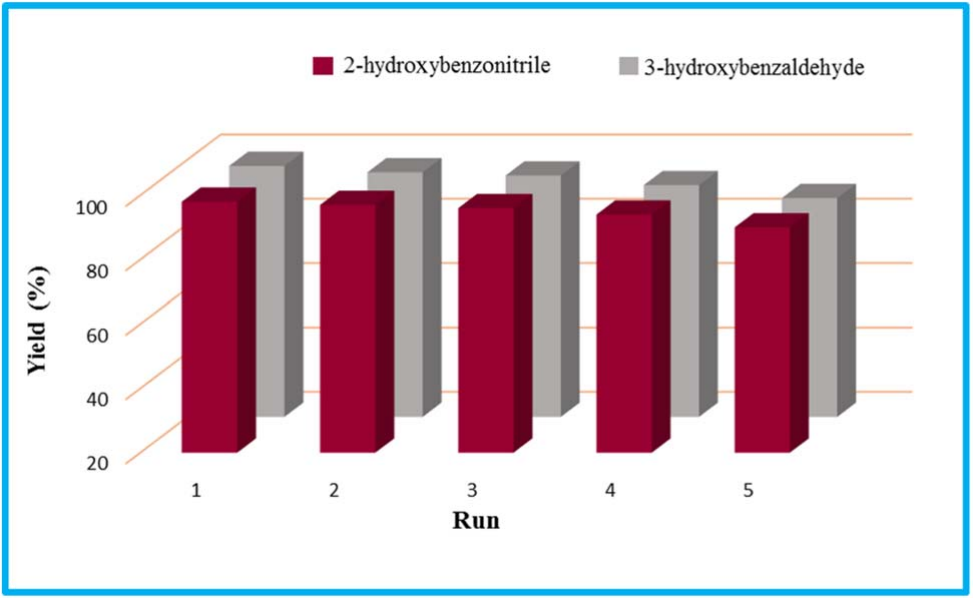
Recyclability study of the KIT-6@SMTU@Ni catalyst in the model tetrazole and pyranopyrazole reactions.

Regarding the final step, the retrievability and reusability of the KIT-6@SMTU@Ni catalyst were investigated in the synthesis of 5-(2-hydroxyphenyl)-1*H*-tetrazole and 6-amino-3-methyl-4-(3-hydroxyphenyl)-2,4-dihydropyrano[2,3-*c*]pyrazole-5-carbonitrile as the model reactions (Fig. 9).

**Fig. 10:**
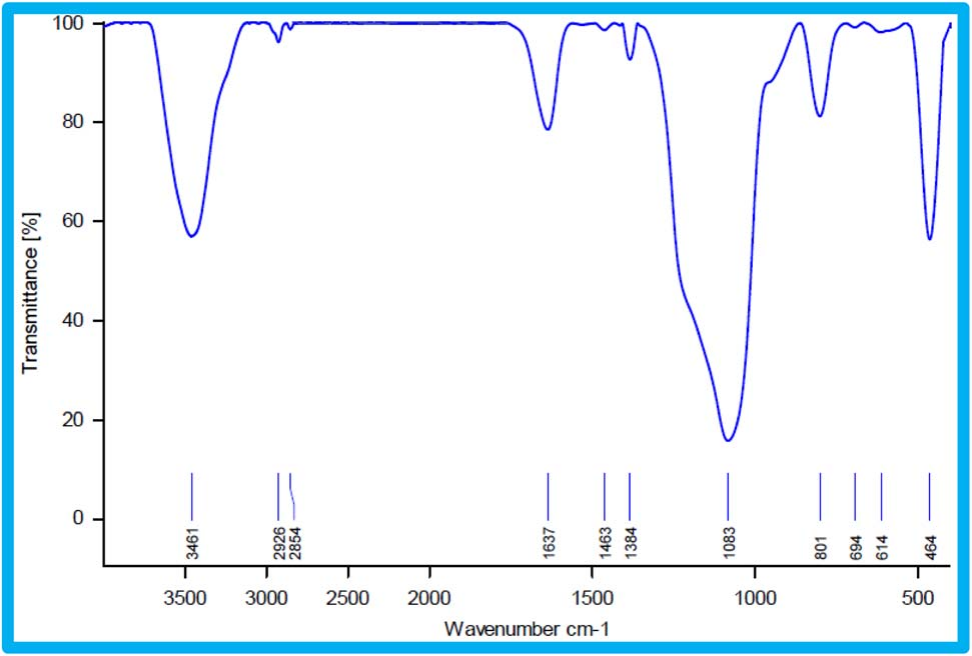
FT-IR spectra of recovered KIT-6@SMTU@Ni.

After finishing the reaction, the catalyst was separated, washed with hot ethyl acetate, dried at 60 °C and then recycled for the subsequent reaction run. The catalyst can be recycled over 5 runs without considerable loss in its activity. The FT-IR spectrum of the recycled catalyst after five cycles does not show any considerable change, compared to the fresh catalyst, which is evidence for the chemical structure of the catalyst remaining stable during the reaction (Fig. 10).

### 3.9 Comparison of the catalyst

As shown in Table 6, to check the performance of KIT-6@SMTU@Ni as the catalyst for the production of the substituted 1*H*-tetrazoles and pyranopyrazoles, the obtained results were compared to the previously reported results of other catalytic systems in the literature. The attractive features of this newly proposed catalyst are short reaction times, recoverability, high reaction yield, recyclability by simple filtration, available and inexpensive starting materials and lack of toxicity.

**Table 6:** Comparison of the KIT-6@SMTU@Ni catalyst for synthesizing 5-phenyl-1*H*-tetrazole with previously reported catalysts

Entry	Catalyst	Time (h)	Yield (%)	Reference
1	KIT-6@SMTU@Ni	3	90	This work
2	CoY zeolite	14	95	71
3	Cu–Zn alloy nanopowder	10	94	72
4	B(C_6_F_5_)_3_	8	95	73
5	Fe_3_O_4_@SiO_2_/salen Cu(ii)	7	81.1	74
6	Fe_3_O_4_/ZnS HNSs	24	86	75
7	Mesoporous ZnS	36	98	76
8	CuFe_2_O_4_	12	90	77
9	Nano ZnO/Co_3_O_4_	12	98	78
10	Cu–TBA@biochar	7	98	7
11	Fe_3_O_4_@boehmite NPs	4	97	79
12	Ni-MP(AMP)_2_@Fe–biochar	4	97	36

## 4 Conclusions

In this study, the synthesis of an SMTU@Ni complex immobilized onto the surface of mesoporous KIT-6 as a new, reusable, and efficient catalyst has been presented. The structure of the catalyst was studied using XRD, EDX, TGA analysis, BET measurements and SEM, and FT-IR spectroscopy. The SEM images of the samples show that the particles are spherical with sizes of about 30 nm for KIT-6 and 40 nm for KIT-6@SMTU@Ni. The XRD pattern shows that the tridimensional symmetric cubic structure of the KIT-6 material remains unchanged after Ni(ii) modification. Moreover, the EDX spectra of the synthesized catalyst show the presence of silicon, oxygen, carbon, nitrogen and also nickel species. The FT-IR analysis proved the presence of Ni(ii) species in the framework of mesoporous KIT-6. The BET studies show that the incorporation of nickel into the silica walls decreased the surface area and pore volume parameters.

Moreover, its catalytic activity was investigated in two important syntheses of tetrazoles using nitrile, NaN_3_, and PEG solvent at 120 °C, and pyranopyrazoles using aldehyde, hydrazine hydrate, malononitrile, ethyl acetoacetate at reflux and temperature 80 °C under ethanol : water solvent conditions. High yields of products, an eco-friendly protocol, short reaction time, simple operating procedure, the use of a novel recyclable catalyst and facile separation of the catalyst by simple filtration are additional benefits from this protocol.

## Conflicts of interest

There are no conflicts to declare.

## Supplementary Material

RA-013-D2RA08269A-s001
