# Supplementary material for: Immobilization of Ni(ii) complex on the surface of mesoporous modified-KIT-6 as a new, reusable and highly efficient nanocatalyst for the synthesis of tetrazole and pyranopyrazole derivatives
Source: RSC Adv. 2023 Apr 24;13(18):12572–88. doi: 10.1039/d2ra08269a (PMC10123498; doi:10.1039/d2ra08269a)

Electronic Supplementary Material (ESI) for RSC Advances.
This journal is © The Royal Society of Chemistry 2023

Supplementary Data

Immobilization of Ni (II) complex on the surface of mesoporous modified-KIT-6 as a new, reusable and highly efficient nanocatalyst for the synthesis of tetrazole and pyranopyrazole derivatives

Mitra Darabi, Mohsen Nikoorazm, Bahman Tahmasbi, Arash Ghorbani-Choghamarani

[a]*Department of Chemistry, Faculty of Science, Ilam University, P. O. Box 69315516, Ilam, Iran.*
[*] *E-mail: e_nikoorazm@yahoo.com*
[b]*Department of Organic Chemistry, Faculty of Chemistry, Bu-Ali Sina University, Hamedan, 6517838683, Iran.*

ABSTRACT: In this paper, KIT-6@SMTU@Ni was successfully synthesized *via* a new Ni (II) complex stabilization on the modified mesoporous KIT-6, as a novel and green heterogeneous catalyst. The obtained catalyst (KIT-6@SMTU@Ni) was characterized using Fourier transform infrared spectroscopy (FT-IR), Brunauer- Emmett- Teller (BET), X-Ray diffraction (XRD), atomic absorption spectroscopy (AAS), energy-dispersive X-ray spectroscopy (EDS), X-ray mapping, thermogravimetric analysis (TGA) techniques and scanning electron microscopy (SEM). After complete characterization of the catalyst, it was successfully used for the synthesis of 5-substituted 1*H*-tetrazoles and pyranopyrazoles. Moreover, tetrazoles were synthesized from benzonitrile derivatives and sodium azide (NaN_3). All tetrazole products were synthesized with high TON, TOF and excellent yields (88-98 %) and reasonable time (0.13-8.08 h), which shows the efficiency and practically of KIT-6@SMTU@Ni catalyst. Furthermore, pyranopyrazoles were prepared through the condensation reaction of benzaldehyde derivatives with malononitrile, hydrazine hydrate and ethyl acetoacetate with high TON, TOF and excellent yields (87-98 %) and appropriate time (2-10.5 h). KIT-6@SMTU@Ni catalyst can reused again for five runs without any re-activation. Significantly, this plotted protocol has prominent benefits such as applying green solvents, use of commercially available and low-cost low cost materials, excellent separation and reusability of the catalyst, short reaction time, high yield of products and facile work-up.

Keywords: mesoporous silica, KIT-6, nanocatalyst, tetrazoles, pyranopyrazoles.

^{1}H NMR spectral data

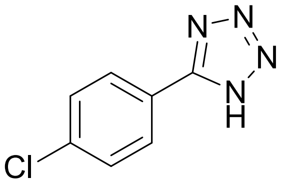

5-(4-chlorophenyl)-1H-tetrazole

^{1}H NMR (400 MHz, $DMSO_{d6}$): δ_H= 8.07-8.04 (d, *J*= 12 Hz, 2H), 7.71-7.68 (d, *J*= 12 Hz, 2H) ppm.

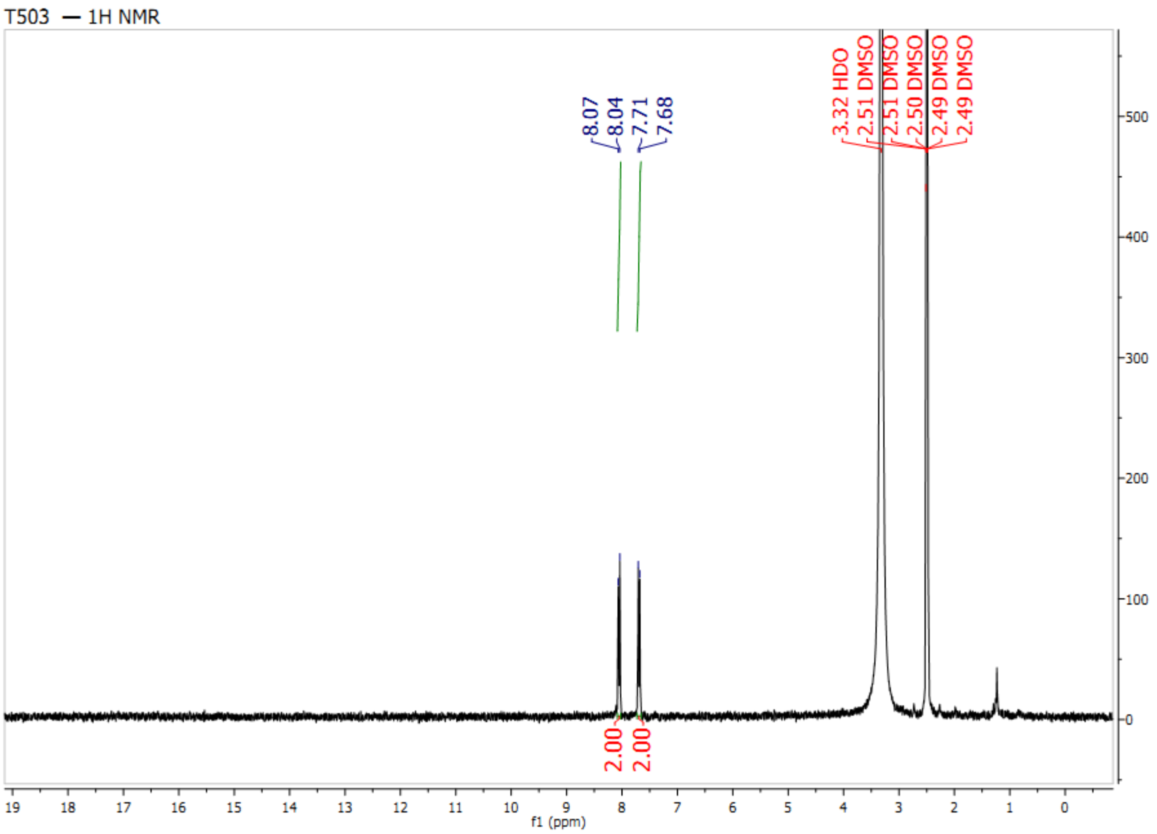

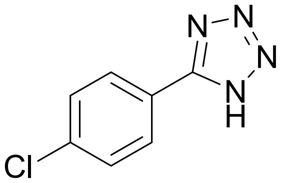

5-(4-chlorophenyl)-1H-tetrazole

^{13}C NMR (100 MHz, DMSO$_{d6}$): δ_H= 164.2, 135.9, 129.6, 128.7, 123.2 ppm.

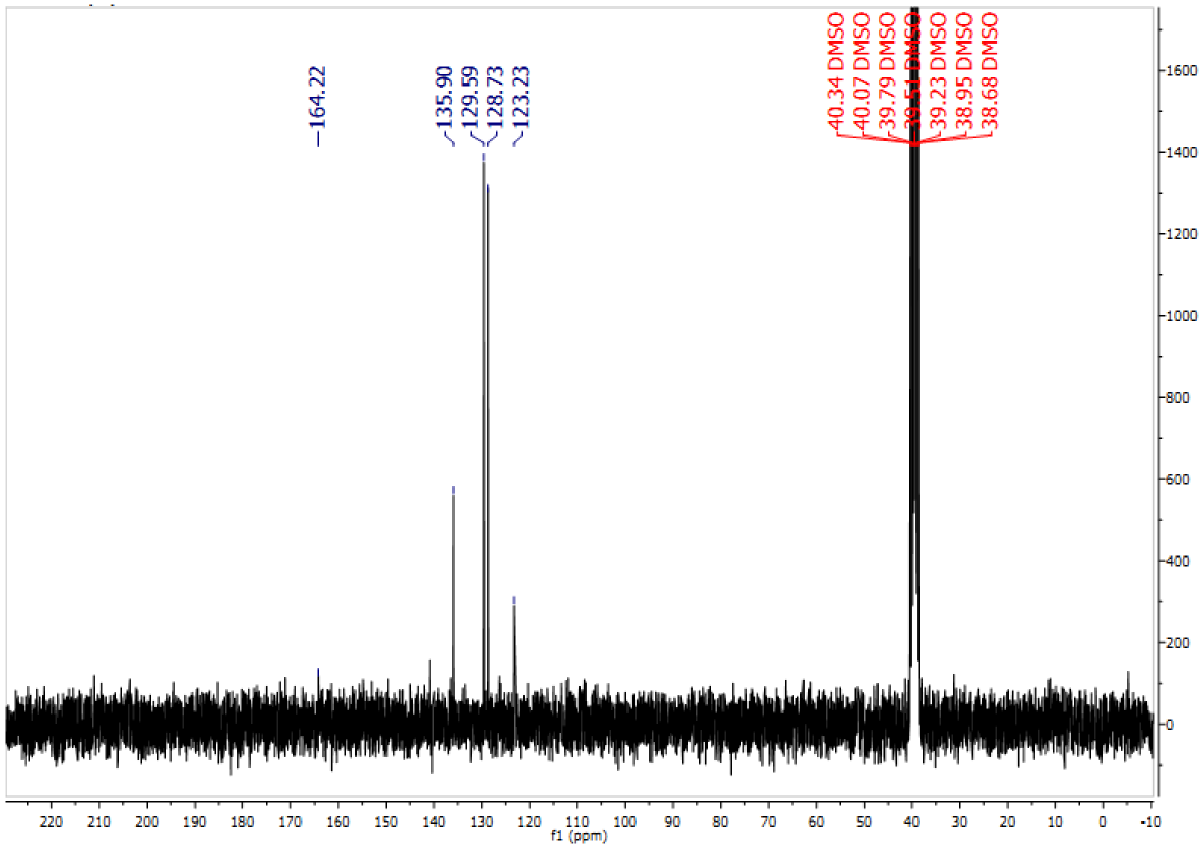

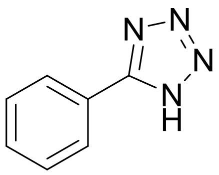

5-phenyl-1H-tetrazole

^{1}H NMR (400 MHz, DMSO$_{d6}$): δ_H= 8.07-8.01 (m, 2H), 7.65-7.58 (m, 3H) ppm.

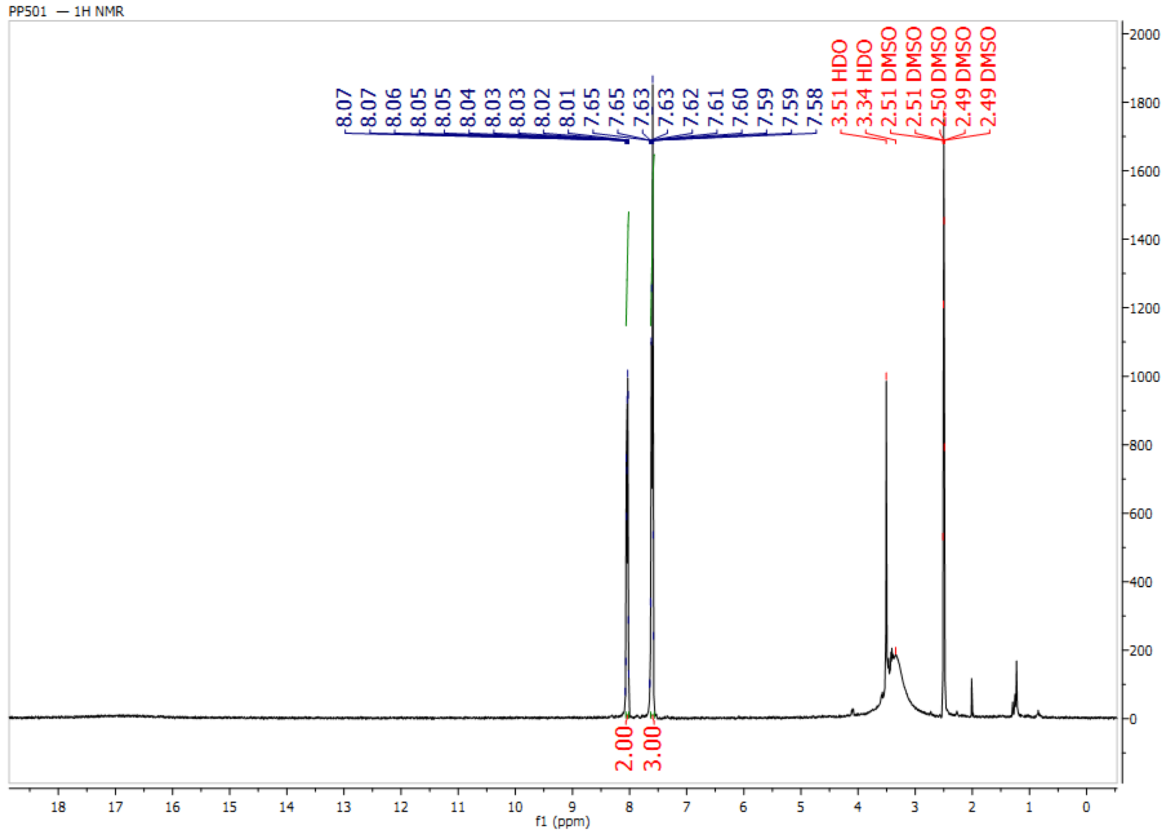

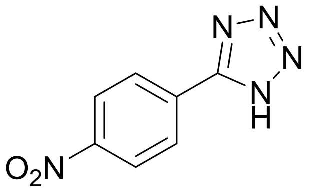

5-(4-nitrophenyl)-1H-tetrazole

^{1}H NMR (400 MHz, DMSO$_{d6}$): δ_H= 8.46-8.43 (d, *J*= 12 Hz, 2H), 8.32-8.29 (d, *J*= 12 Hz, 2H) ppm.

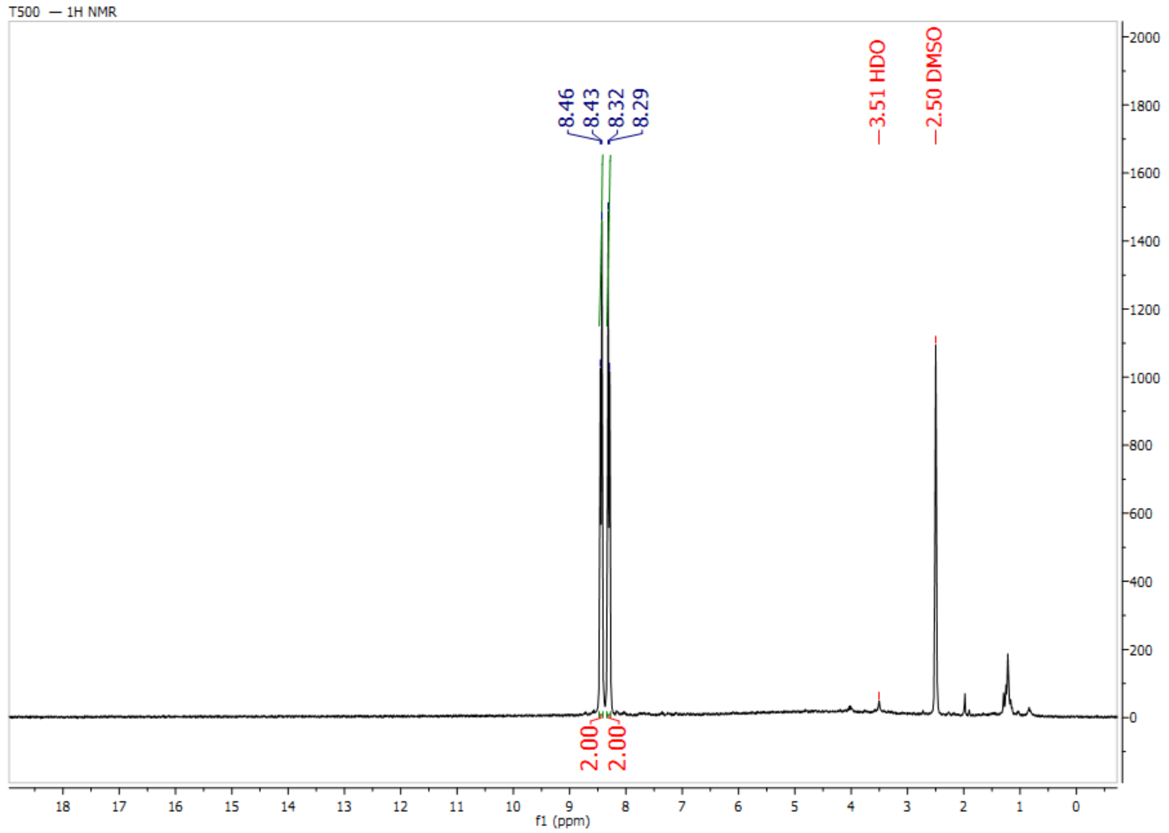

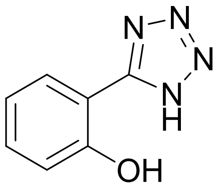

2-(1H-tetrazol-5-yl)phenol

^{1}H NMR (400 MHz, $DMSO_{d6}$): δ_H= 8.00-7.97 (d, *J*= 12 Hz, 1H), 7.44-7.38 (t, *J*= 12 Hz, 1H), 7.09-7.06 (d, *J*= 12 Hz, 1H), 7.03-6.98 (t, *J*= 12 Hz, 1H), 3.36 (br, 1H) ppm.

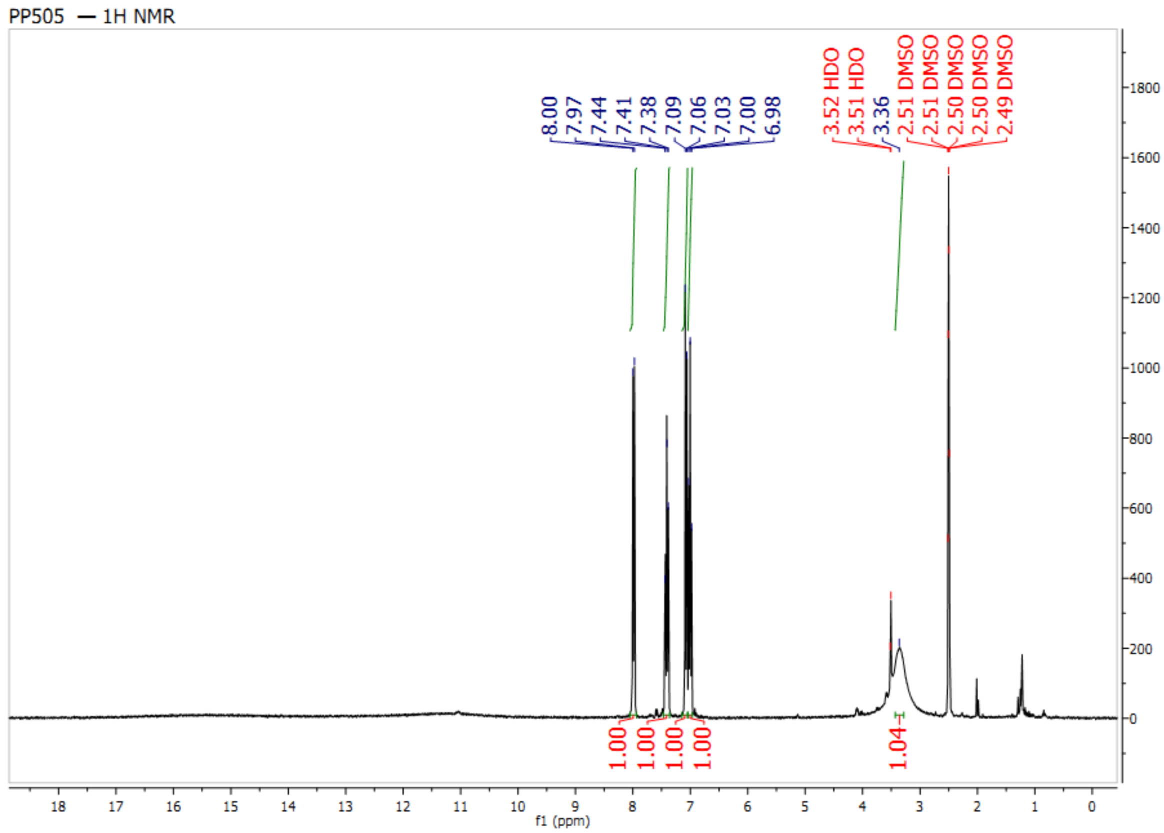

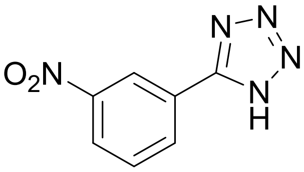

5-(3-nitrophenyl)-1H-tetrazole

^{1}H NMR (400 MHz, $DMSO_{d6}$): δ_H= 8.83 (s, *1*H), 8.48-8.46 (d, *J*= 8 Hz, 1H), 8.42-8.40 (d, *J*= 8 Hz, 1H), 7.93-6.87 (t, *J*= 12 Hz, 1H) ppm.

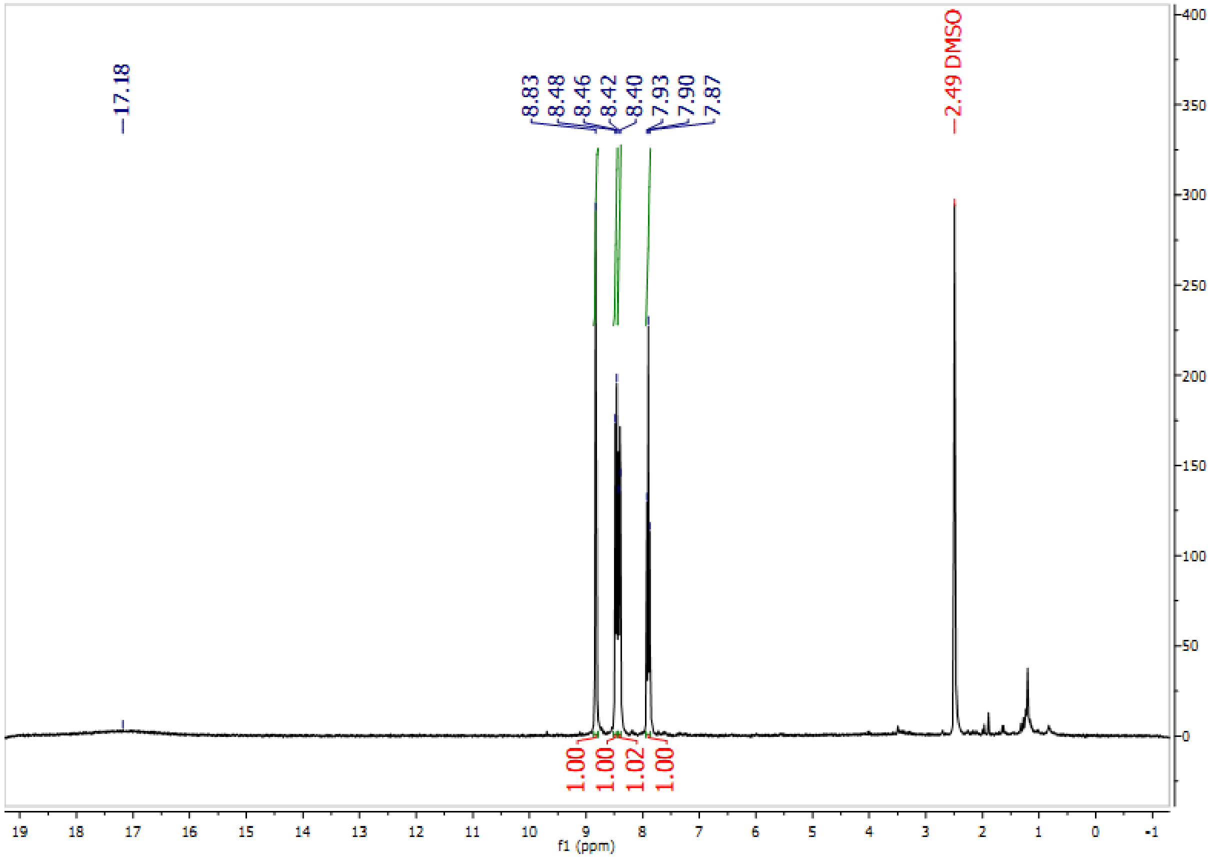

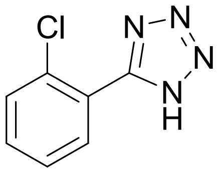

5-(2-chlorophenyl)-1H-tetrazole

^{1}H NMR (400 MHz, DMSO$_{d6}$): δ_H= 16.93 (br, 1H), 7.82-7.79 (d of d, *J*= 8 Hz, 1H), 7.72-7.69 (d, *J*= 8 Hz, 1H), 7.65-7.60 (t of d, *J*= 8 Hz, 1H), 7.58-7.53 (t of d, *J*= 8 Hz, 1H) ppm.

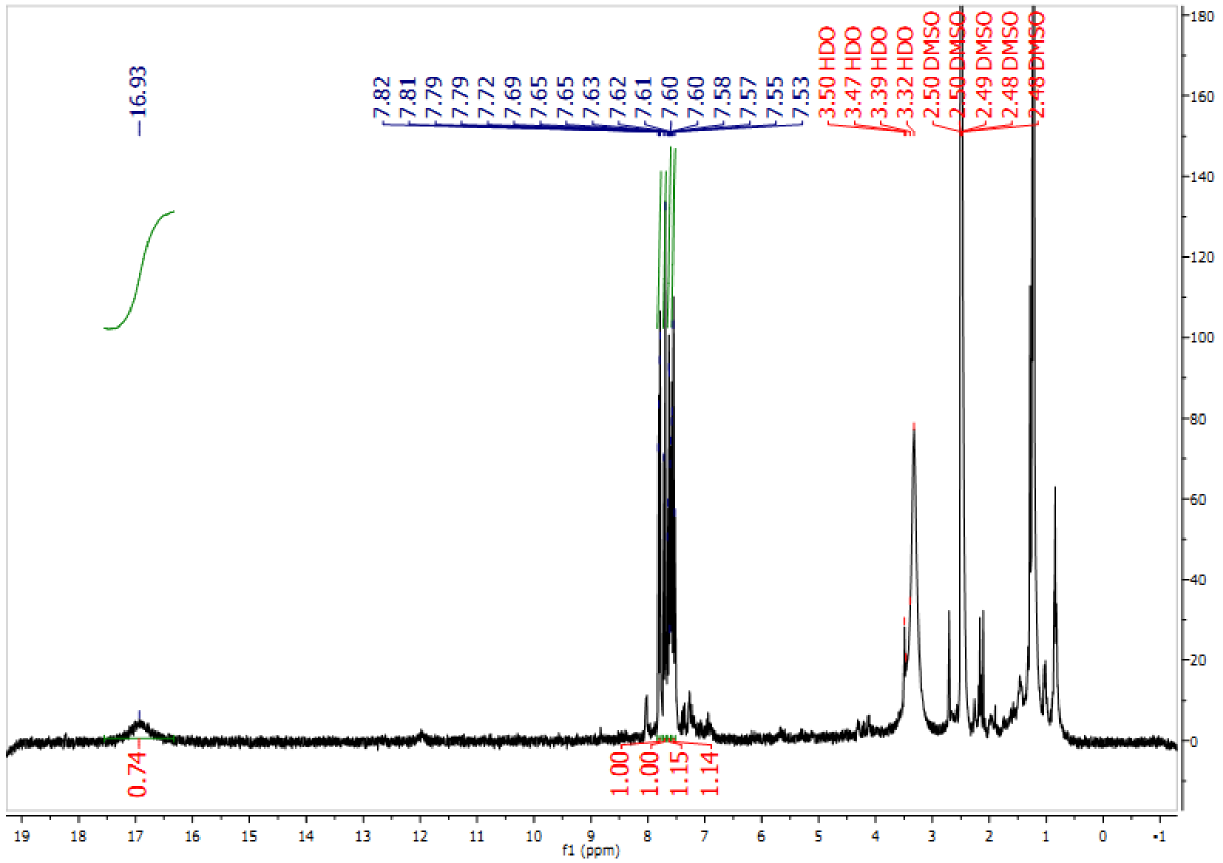

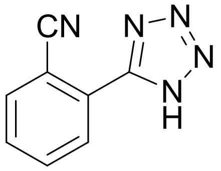

2-(1H-tetrazol-5-yl)benzonitrile

^{1}H NMR (400 MHz, $DMSO_{d6}$): δ_H= 8.11-8.06 (t, *J*= 8 Hz, 2H), 7.95-7.90 (t, *J*= 8 Hz, 1H), 7.81-7.75 (t, *J*= 8 Hz, 1H) ppm.

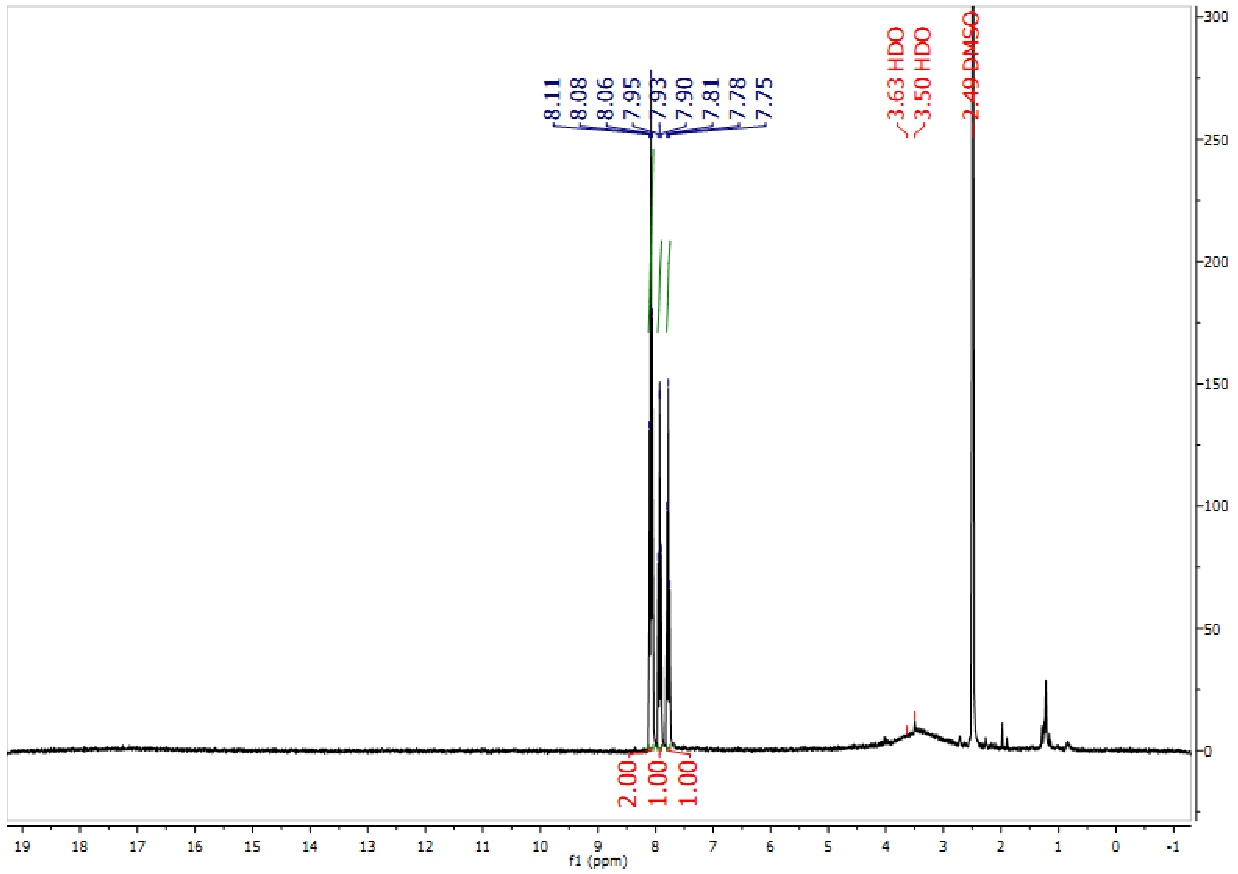

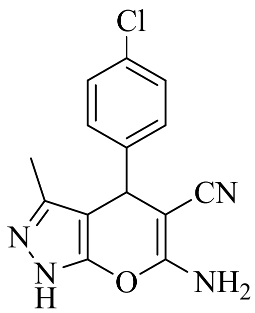

6-amino-4-(4-chlorophenyl)-3-methyl-1,4-dihydropyrano[2,3-c]pyrazole-5-carbonitrile

^{1}H NMR (400 MHz, $DMSO_{d6}$): δ_H= 12.09 (s, 1H), 7.64-7.52 (m, 2H), 7.40-7.32 (m, 2H), 6.98 (br, 2H), 5.57 (s, 1H), 1.77 (s, 3H) ppm.

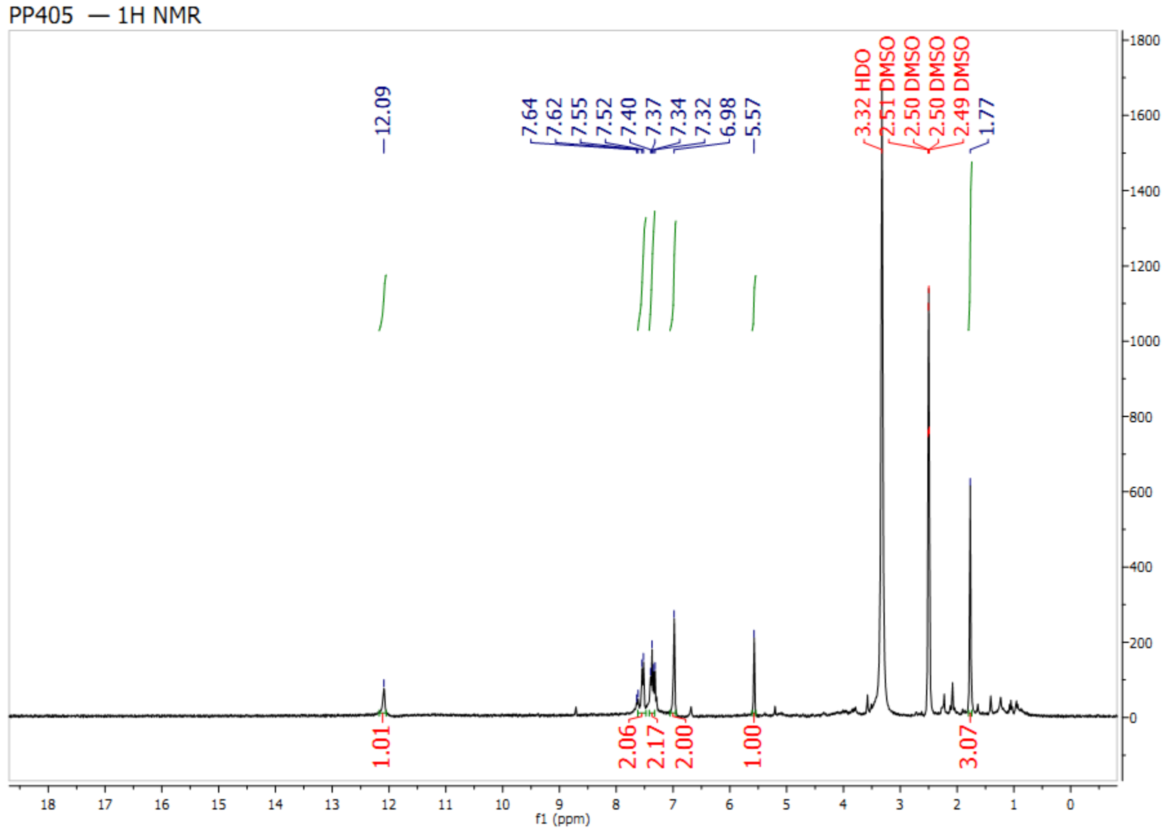

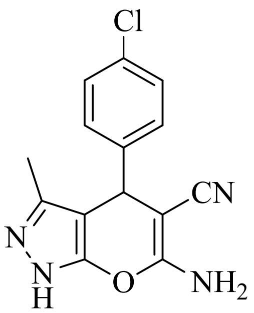

6-amino-4-(4-chlorophenyl)-3-methyl-1,4-dihydropyrano[2,3-c]pyrazole-5-carbonitrile

^{13}C NMR (100 MHz, DMSO$_{d6}$): δ_H= 160.9, 154.7, 143.5, 135.7, 131.2, 130.0, 128.5, 120.7, 97.2, 56.7, 35.5, 9.7 ppm.

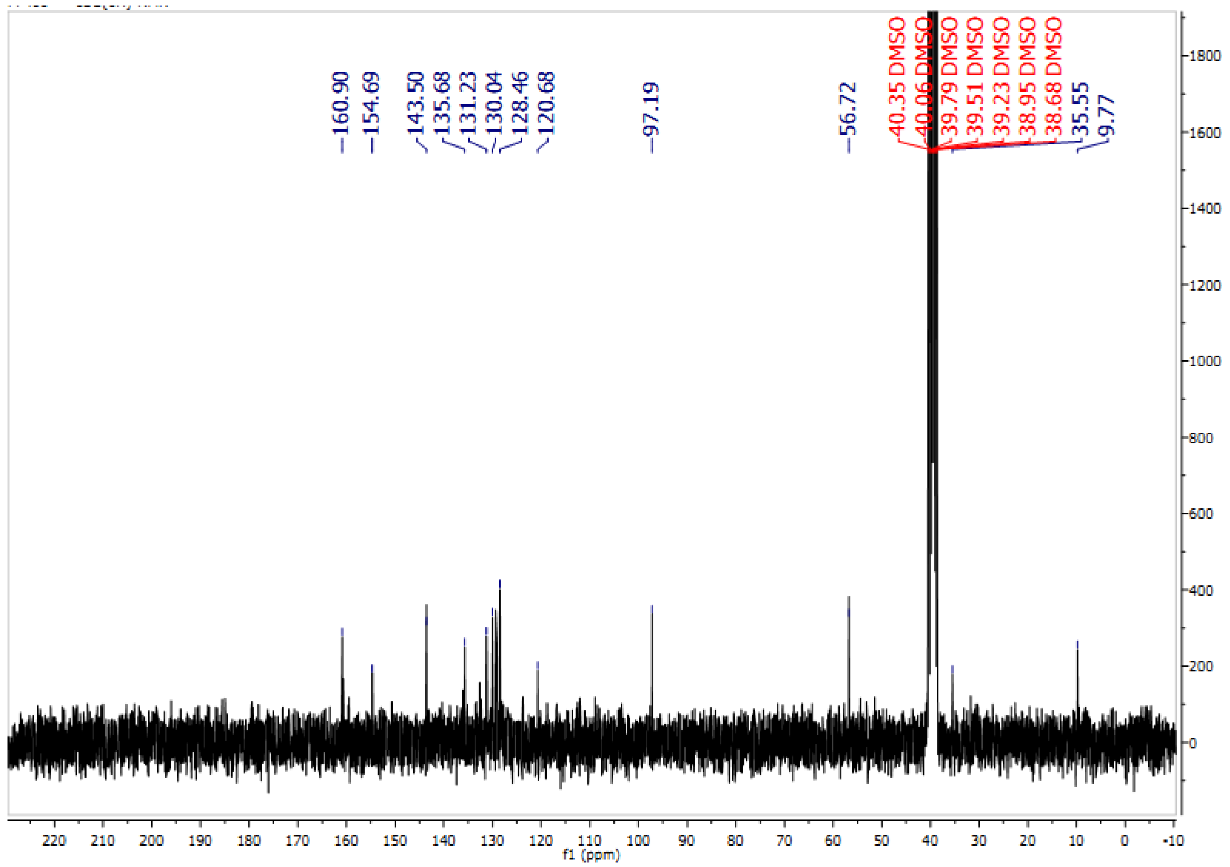

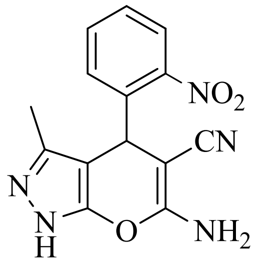

6-amino-3-methyl-4-(2-nitrophenyl)-1,4-dihydropyrano[2,3-c]pyrazole-5-carbonitrile

^{1}H NMR (400 MHz, DMSO$_{d6}$): δ_H= 12.20 (s, 1H), 7.86-7.84 (d, *J*= 8 Hz, 1H), 7.69-7.64 (t, *J*= 12 Hz, 1H), 7.52-7.46 (t, *J*= 12 Hz, 1H), 7.33-7.31 (d, *J*= 8 Hz, 1H), 7.02 (s, 2H), 5.09 (s, 1H), 1.77 (s, 3H) ppm.

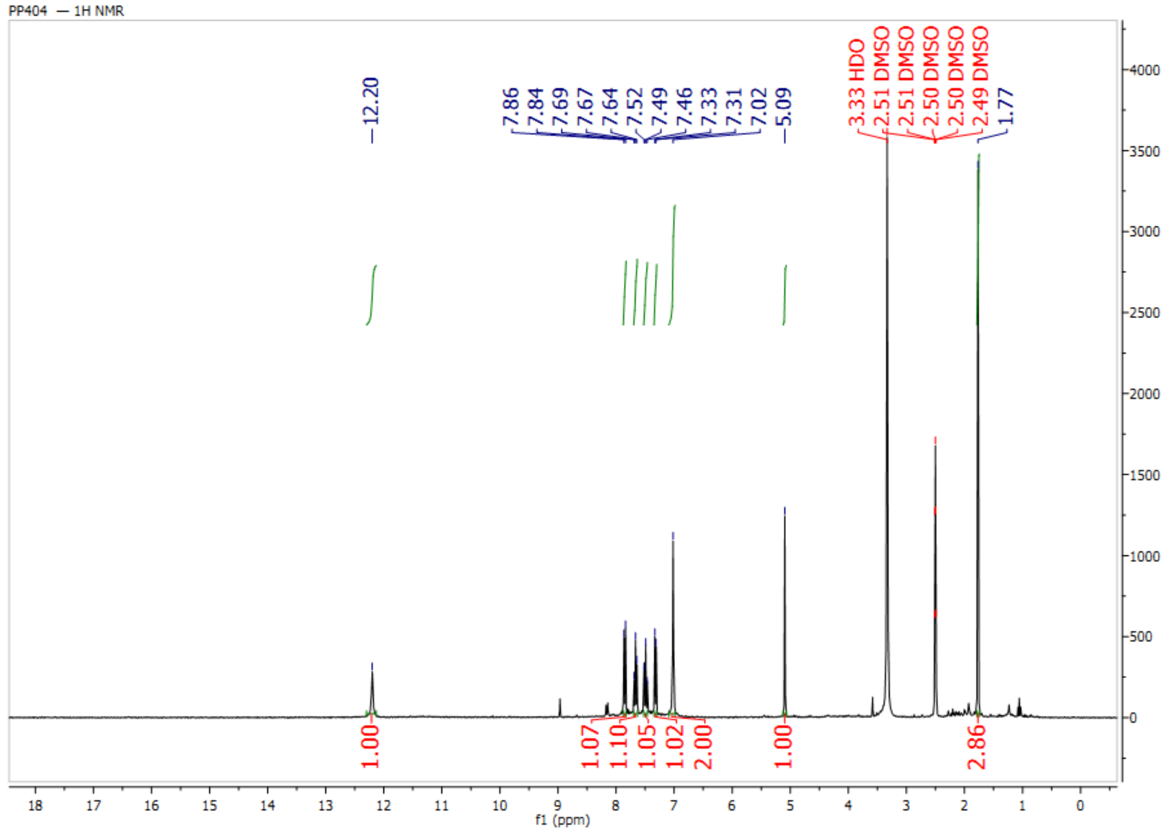

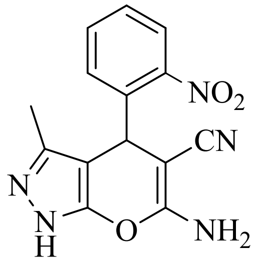

6-amino-3-methyl-4-(2-nitrophenyl)-1,4-dihydropyrano[2,3-c]pyrazole-5-carbonitrile

^{13}C NMR (100 MHz, DMSO$_{d6}$): δ_H= 161.1, 154.9, 149.1, 137.6, 135.7, 131.3, 128.3, 123.6, 120.2, 96.4, 55.9, 31.4, 90.5 ppm.

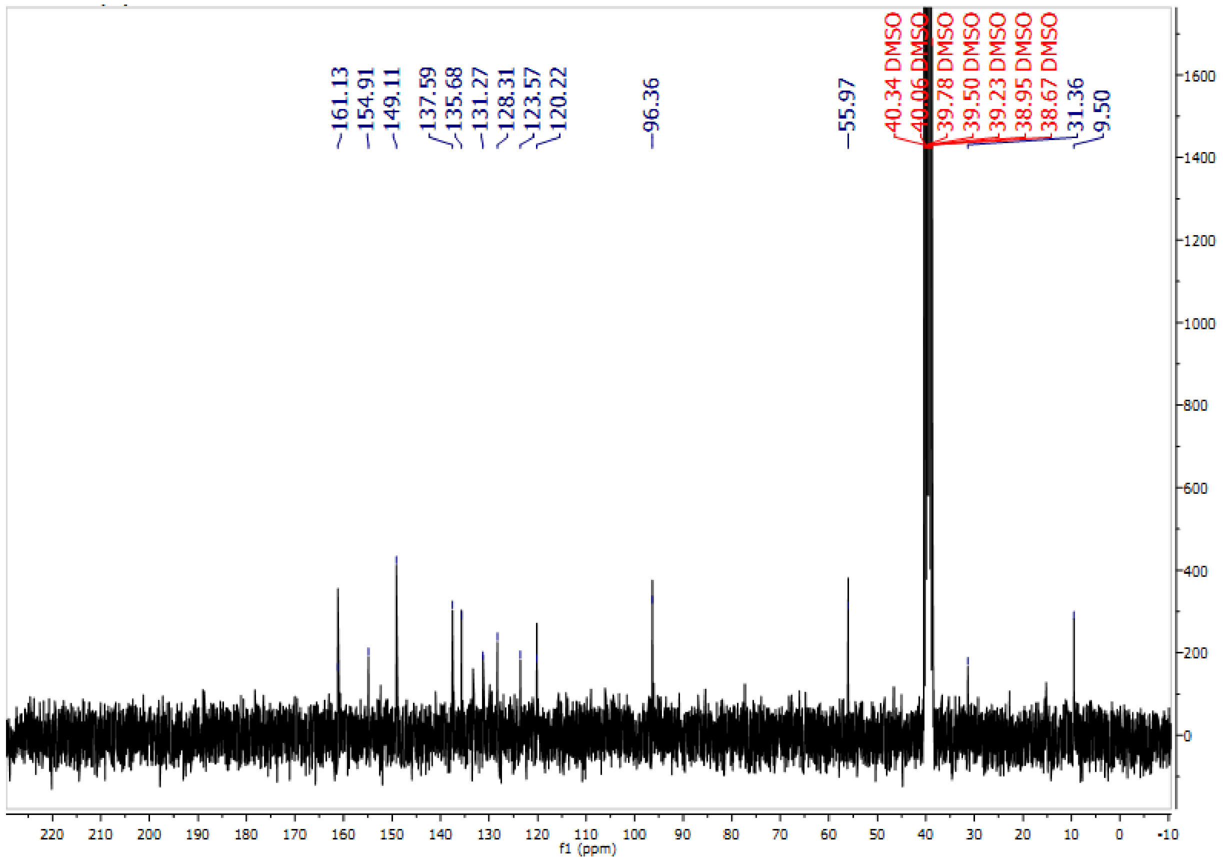

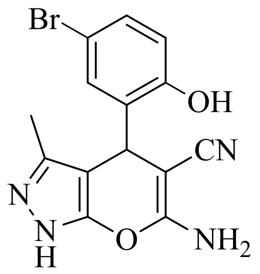

6-amino-4-(5-bromo-2-hydroxyphenyl)-3-methyl-1,4-dihydropyrano[2,3-c]pyrazole-5-carbonitrile

^{1}H NMR (400 MHz, DMSO$_{d6}$): δ_H= 11.16 (br, 1H), 8.93 (s, 1H), 7.36-7.32 (d, J= 16 Hz, 1H), 7.12 (s, 1H), 6.95-6.91 (d, J= 16 Hz, 1H), 6.78 (br, 2H), 4.62 (s, 1H), 3.33 (s, 1H), 2.01 (s, 3H) ppm.

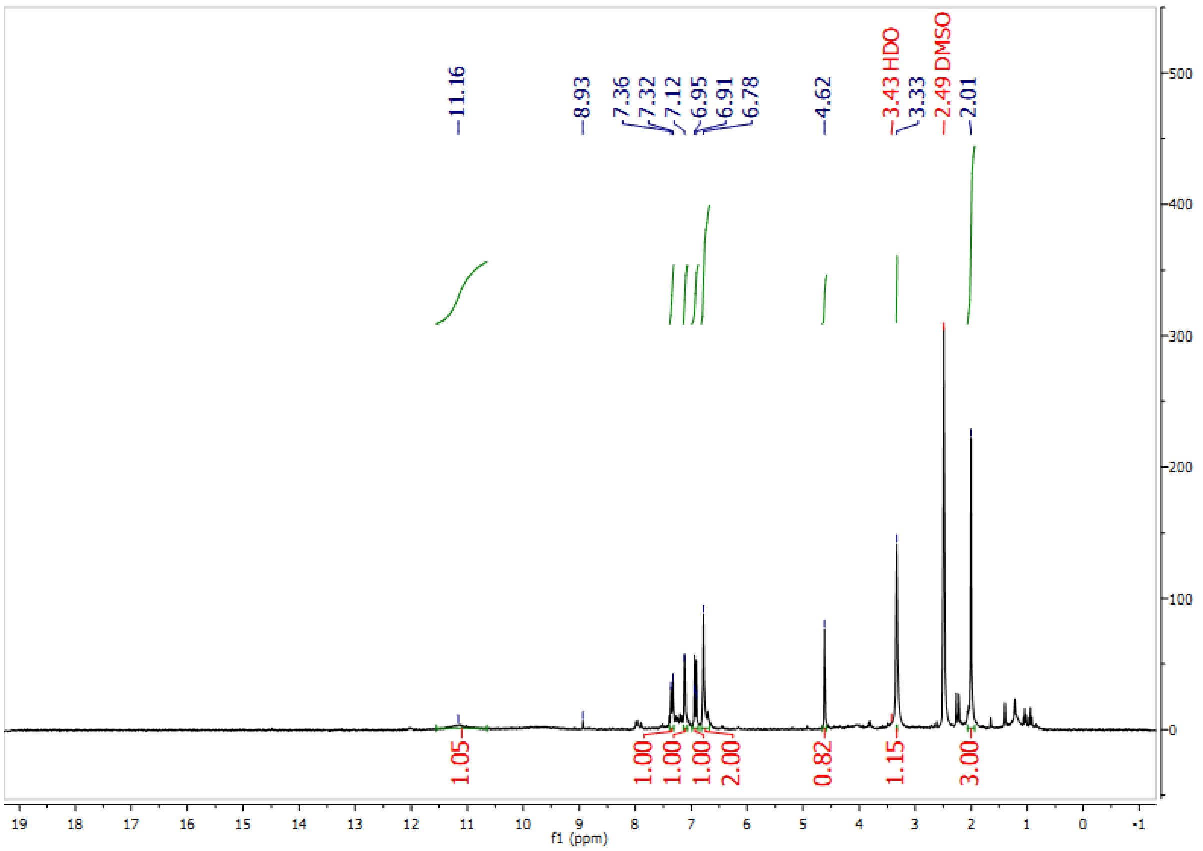

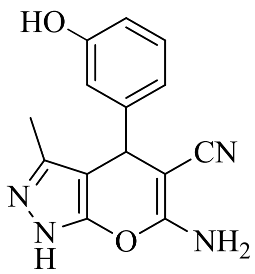

6-amino-4-(3-hydroxyphenyl)-3-methyl-1,4-dihydropyrano[2,3-c]pyrazole-5-carbonitrile

^{1}H NMR (400 MHz, DMSO$_{d6}$): δ_H= 12.09 (br, 1H), 9.32 (s, 1H), 7.11-7.06 (d, *J*= 8 Hz, 1H), 6.86 (br, 2H), 6.62-6.58 (m, 2H), 6.52 (s, 1H), 4.47 (s, 1H), 3.36 (br, 1H), 1.81 (s, 3H) ppm.

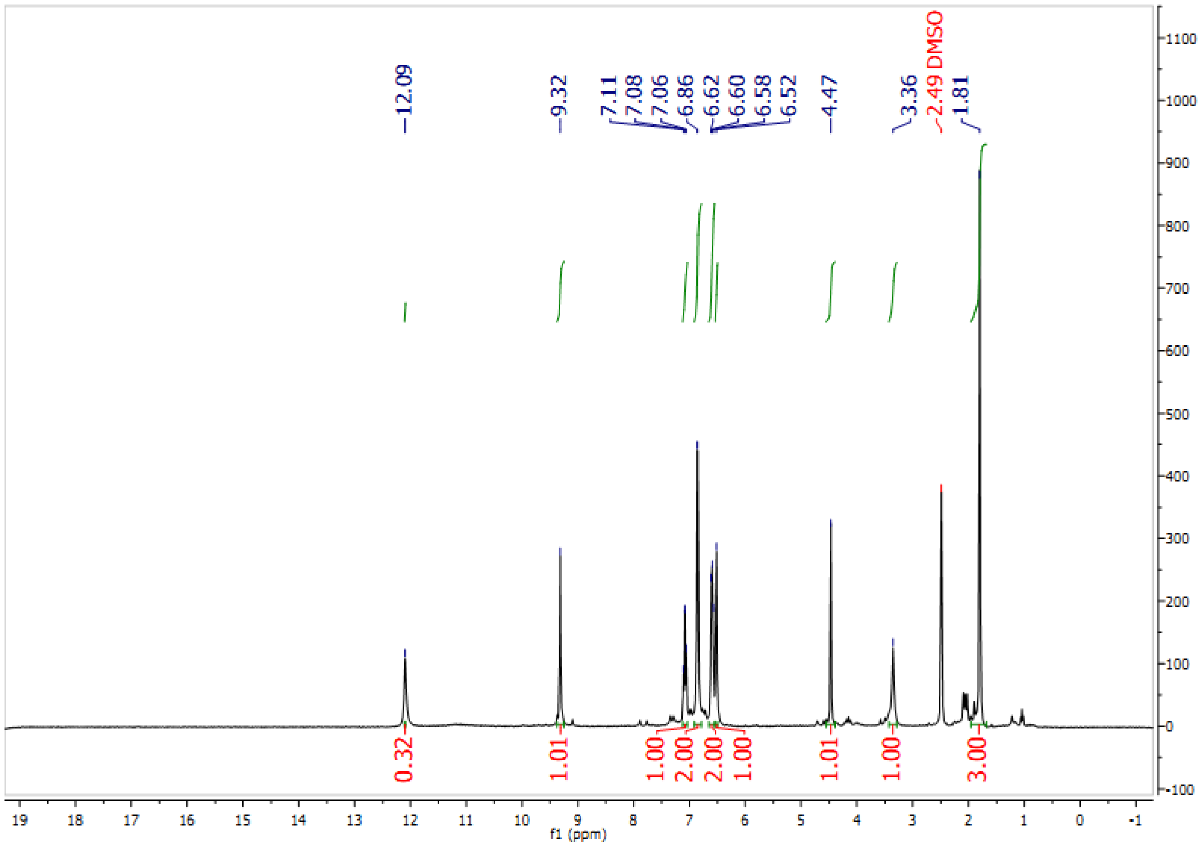

Supplement: RA-013-D2RA08269A-s001 [file RA-013-D2RA08269A-s001.pdf]
